# Optimized multi-frequency nonlinear broadband piezoelectric energy harvester designs

**DOI:** 10.1038/s41598-024-61355-1

**Published:** 2024-05-18

**Authors:** Mohamed A. Elgamal, Hassan Elgamal, Sallam A. Kouritem

**Affiliations:** Department of Mechanical Engineering, Faculty of Engineering, Alexandria University, Alexandria, 21544 Egypt

**Keywords:** Piezoelectric energy harvesting, Broadband natural frequency, Optimization, FEA, Nonlinear designs, Mechanical engineering, Energy grids and networks

## Abstract

Many electrical devices can be powered and operated by harvesting the wasted energy of the surroundings. This research aims to overcome the challenges of output power with a sharp peak, small bandwidth, and the huge dimensions of the piezoelectric energy harvesters relative to the output power. The aforementioned challenges motivated us to investigate the effect of nonlinearity in the shape (tapered and straight cross-section area) as well as the fixation method (the number of fastened ends) to determine the optimal design with high output power and wide working frequency. This research proposes a novel piezoelectric energy harvester array, where each beam is made up of three fixed beams that are joined together by a center mass. The proposed design produces an output power of 35 mW between 25 and 40 Hz. The output power of the proposed design is 3.24 times more than the conventional designs. The recommended approach is simulated utilizing finite element analysis FEA. Analytical and experimental methods validate the proposed FEA, which exhibits excellent agreement.

## Introduction

**Figure 1:**
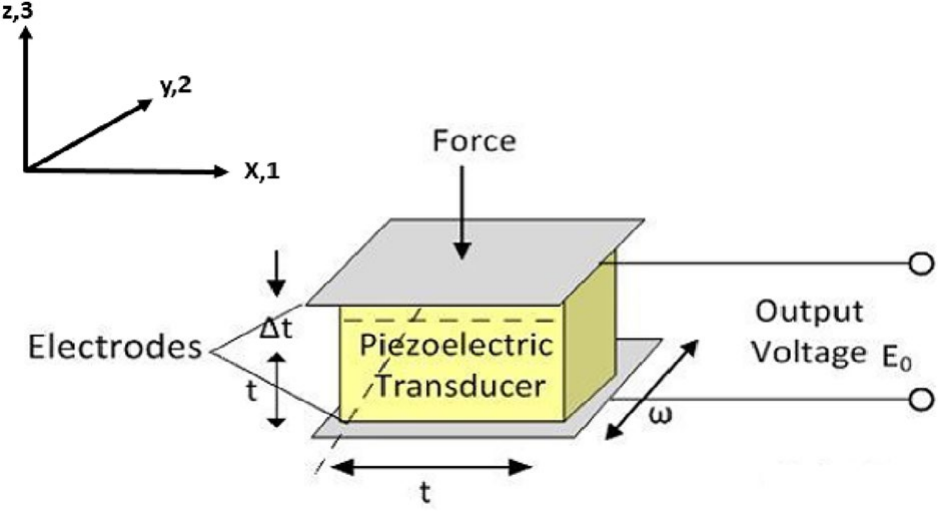
Schematic drawing of a piezoelectric transducer, where axis 3 represents the piezoelectric material's initial polarization^14^.

The need for energy in society is growing as a result of population growth and global economic growth^1^. Countries are pushing for green energy initiatives to achieve sustainable development, as traditional sources fall short. Solar, thermal, RF, ocean waves, wind, and mechanical vibration energy are common examples^2–5^. Over the past 10 years, vibration energy harvesting devices have received a lot of interest. As a result, significant research efforts have been made. Based on various conversion techniques, numerous researchers have tried to create some mechanical-to-electrical energy systems^6–10^. Because it can continually supply energy for low-power wireless sensor networks and microelectronic systems, vibration energy collection is favored by researchers^11,12^. Linear resonance is the principle of collecting used by the majority of modern energy harvesting systems. The energy conversion efficiency is highest when the ambient vibration source's vibration frequency is close to the natural frequency of the capture device. The efficiency of the energy-capturing device rapidly declines as it departs from the natural frequency^13^. Piezoelectric transduction has drawn a lot of interest as one of the crucial vibration-based energy harvesting techniques due to the straightforward design of the piezoelectric generator and the practicality of piezoelectric materials. The direct piezoelectric effect is the process of using a piezoelectric transducer to convert mechanical strain into an electrical charge. Typically, the ambient vibration in the area around the power harvesting equipment is what causes the mechanical strain. Low-level energy, on the scale of microwatts to milliwatts, is typically the focus of piezoelectric energy harvesting of ambient vibration. Figure 1 shows the schematic diagram of a piezoelectric transducer, in which axis 3 denotes the initial polarization of the piezoelectric material.

The researchers have worked very hard to broadband the natural frequency range of energy harvesting devices, hence over the past ten years, many nonlinear and broadband energy harvesting approaches have been developed and explored to boost the efficiency of linear systems' energy harvesting by expanding the harvester's response bandwidth. Goldschmidtboeing and Woias^15^ analyzed various beam designs, including triangular and rectangular shapes, focusing on efficiency and maximum excitation amplitude. They found that beam shape significantly influences the maximum excitation amplitude and, consequently, the maximum output power, with little impact on efficiency. Hwang et al.^16^ developed a piezoelectric tile for energy harvesting from footsteps, aiming to optimize energy capture. To prevent damage to piezoelectric modules from direct exposure to human movement energy, they employed a tile design using indirect energy transmission via springs and a tip mass.

Ramalingama et al.^17^ designed a piezoelectric energy harvester that uses two beam configurations with varying cross sections and tapered cavities to enhance the broadband frequency. Izadgoshasb et al.^18^ introduced a cantilever beam with a piezoelectric patch attached at one end to examine the viability of harvesting energy from human motion. Li and Jing^19^ constructed a nonlinear x-shaped structure coupled to piezoelectric harvesters through two types of specific mounting configurations (horizontal and vertical cases), to use structural coupling and nonlinearity in vibration energy harvesting. Wang et al.^20^ presented the design and fabricating process of a packaged micro piezoelectric vibration energy harvester (PVEH). They found that output power is 10 times more than that of the conventional L-shape due to thick copper-based PZT bimorph and double L-shaped tungsten-proof mass blocks that ensure its high electrical power. Li et al.^21^ proposed a generalized multi-mode PEH (MPEH) accompanied by analytical modeling. The suggested MPEH consists of a primary cantilevered beam attached to several branches with tip masses at their free ends and bound by a patch of piezoelectric material. Jia et al.^22^ involved the dynamic mechanical and electrical behavior of piezoelectric macro fiber composite (MFC) on carbon fiber composite beams. Additionally, they looked into how the energy harvesting capabilities made feasible by MFC integration would aid the aerospace, automotive, and renewable energy industries. Sun and Tse^23^ exploited a new horizontal asymmetric U-shaped vibration-based piezoelectric energy harvester (U-VPEH), which gathers and transforms harmful vibration energy into beneficial electrical energy. Dhote et al.^24^ compared experimentally different nonlinear multi-frequency compliant ortho planar spring types with numerous piezoelectric plates for energy harvesting. The COPS designs for the bi-leg, tri-leg, quad-leg, and penta-leg have all been developed and rigorously tested. The most effective of them is the quad-leg design. It offers forward and reverse sweeps with a continuous nonlinear voltage response. Asthana and Khanna^25^ simulated and analyzed a wide-band low-frequency piezoelectric energy harvester that can capture the greatest amount of environmental vibrations. Due to its small size the device can be used for several purposes. Ma et al.^26^ suggested to use a Z-type broadband piezoelectric cantilever energy harvester. Additionally, it was looked at whether the first-order and second-order modes of the piezoelectric energy harvester have a decreasing natural frequency spacing with increasing beam length. Ghayesha and Farokhi^27^ studied the performance of a nonlinear constrained bimorph piezoelectric energy harvester under parametric excitation. An energy harvester that captures the energy of fundamental parametric motions was presented in two different designs. The employment of stoppers and an extra tip mass in combination with parametric excitation considerably improves the energy harvester's performance. Lu et al.^28^ investigated nonlinear energy harvesting from the transverse vibrations of a two-span beam. To improve the performance, a two-span beam that has buckled and flexed piezoelectric film is used. Li et al.^29^ proposed a bi-stable piezoelectric energy harvester which consists of a piezoelectric cantilever beam with a tip magnet and a movable magnet. The movable magnet is attached to the base by a spring and is free to move in the direction of the spring compression. Qian et al.^30^ designed a bi-stable piezoelectric energy harvester, Inspired by the rapid shape transition of the Venus flytrap. Lia et al.^31^ exploited multi-modal and multi-directional responses of a simple structure. They investigated the possibility of creating broadband and highly effective bending-torsion L-shaped bimorph harvester by exploitation of the ${d}_{31}$ and ${d}_{36}$ modes, given advancements achieved in manufacturing piezoelectric materials with large face shear piezoelectric coefficients (${d}_{36}$). Qian et al.^32^ modeled A broadband piezoelectric energy harvester (PEH) with a mechanically tunable potential function. Both monostable and bistable configurations can be optimized for the harvester, which consists of a beam and a pre-compression spring at one end. Kouritem^33^ introduced three graded cantilevers with six masses to broaden the bandwidth natural frequency. The masses are arranged to create an angle with the Y-axis. Cao et al.^34^ investigated the vibration characteristics of a cantilever L-shaped beam perpendicular to the horizontal plane. It is concluded that the distributed piezoelectric energy harvester is best positioned close to the fixed end of the entire cantilever structure. Jianga et al.^35^ proposed a V-shape folded piezoelectric energy harvester with an impact stopper. Performance evaluations revealed the permissible power density for V-PVEH (V-shaped piezoelectric energy harvester) is 8 times that of C-PVEH (C-shaped piezoelectric energy harvester) with the same bimorph length. Fan et al.^36^ designed a wideband two-element piezoelectric energy harvester with both bi-stability and parametric resonance characteristics, to address the challenge of lowering the potential barrier and triggering the parametric threshold amplitude. Mohamed et al.^37^ constructed Five harvester different designs, namely, the T-shaped, rectangular, L-shaped, variable width, and triangular cantilevers to examine the impact of the harvester characteristics on the output power and efficiency of the harvester. Hani et al.^38^ designed a piezoelectric cantilever with two concentrated masses that have two degrees of freedom (2-DOF). A broad natural frequency (1–41 Hz) is provided by the proposed design. Wang et al.^39^ introduced a multi-folded-beam piezoelectric energy harvester (MFB-PEH) for low-power energy harvesting applications in situations with high frequency, low frequency, and low amplitude vibration. One conventional main beam and two novel, L-shaped wing beams make up the MFB-PEH. Koureitem et al.^40^ adapted the tip masses of the cantilevers to make an angle with the vertical y-axis (α). It was discovered that raising (α) enhances the bandwidth natural frequency as well as the output power. Hani et al.^41^ designed a rainfall droplet impact force sensing device that is composed of a bimorph simply supported composite-piezoelectric beam. In this research, the automated genetic algorithm (GA) optimization technique is used to enhance the observed voltage signal. Dong et al.^42^ developed a novel piezoelectric cantilever for effectively gathering track vibration energy, with a dual mass arrangement and trimly tuned bandwidth. Hani et al.^43^ presented a sensor consisting of an array of 17 individual uniform simply supported composite-PZT beams with a concentrated mass in the mid-span of each beam to convert Earthquake-induced structural vibration into an instant voltage signal via piezoelectric (PZT). Shao et al.^44^ introduced a piezoelectric energy harvesting (PEH) system with two degrees of freedom (2-DOF), incorporating a stopper for broad operation. Their device utilizes mechanical stopper-induced nonlinear dynamics and multimodal energy harvesting techniques, featuring a folded piezoelectric cantilever with integrated stopper. Zhang et al.^45^ proposed a multi-frequency response piecewise-linear piezoelectric vibration energy harvester (MFRPLP-VEH), By integrating the linear multi-frequency resonance and nonlinear vibration bandwidth widening techniques, the piezoelectric vibration energy harvester's operational frequency broadness and environmental robustness can be increased. Fan et al.^46^ developed a nonlinear piezoelectric energy harvesting array with wideband performance under both direct and parametric excitation. The array consists of four cantilever beams, each with attached piezoelectric layers and distinct tip masses.

**Figure 2:**
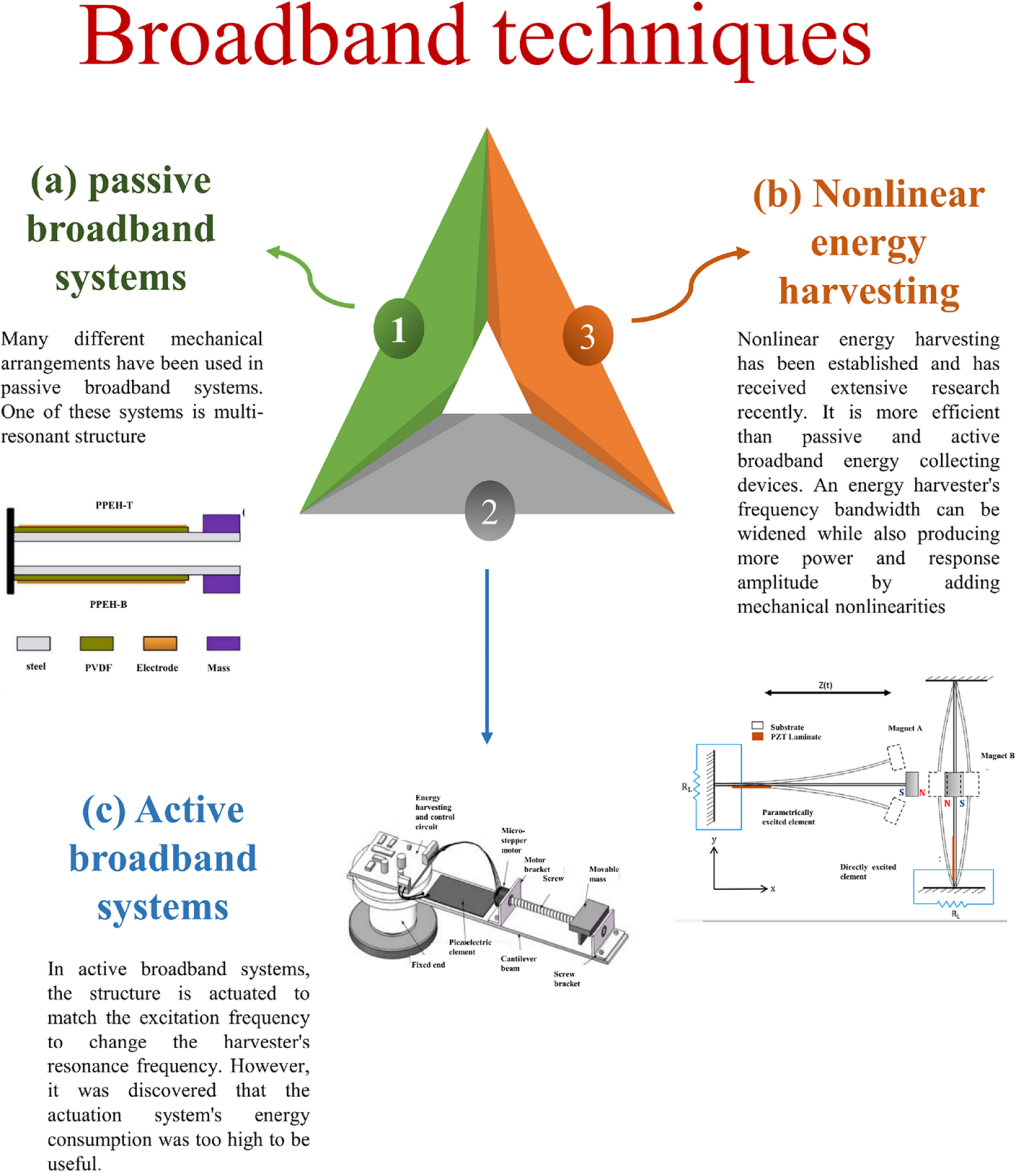
Comparison between the current broadband natural frequency techniques. (**a**) Passive broadband systems, (**b**) nonlinear energy harvesting, (**c**) active broadband systems.

Kouritem et al.^47^ designed an L-shaped harvester with concentrated masses. The findings demonstrate that increasing the number of concentrated masses raises output power and broad natural frequency. Wang et al.^48^ proposed a compact ultralow-frequency and broadband piezoelectric energy harvester (UBPEH). The T-shaped UBPEH uses magnetic interaction to reduce stiffness and improve stopper performance in the low-frequency band. Chen et al.^49^ presented a piezoelectric energy harvester with a magnetic chaotic pendulum. The operating frequency of pendulum energy harvesters is 10 Hz, primarily from human and oceanic motion energy. Li et al.^50^ introduced a piezoelectric–electromagnetic hybrid flutter-based energy harvester (HFEH), where magnetic forces connect the electromagnetic and piezoelectric components. A nonlinear study suggests that the dynamic magnetic coupling force between the piezoelectric and electromagnetic parts could boost the overall power of the HFEH. Zhao et al.^51^ suggested a graded metamaterial-based energy harvester with an emphasis on related vibrations (100 Hz) for broad-band and high-capability piezoelectric energy harvesting. Kouritem and Altabey^52,53^ proposed the Automatic Resonance Tuning (ART) technique of two piezoelectric beams to manage the challenges of output power with a sharp peak, small enhancement in bandwidth, and large dimensions and weights of the harvesters. Therefore, a significant effort was made to extend the harvester's operating frequency range. We attempted to condense the most significant broadband strategies in Fig. 2.

According to the literature, the harvester's design has two weaknesses. First off, many systems lack bandwidth, resulting in output power that has a sharp peak (one harvester). Second, the various solutions that have already demonstrated the bandwidth either have low output power in the operating frequency or require additional components such as a magnet, sensor, actuators, and complex construction (many techniques rely on additional components like active tuning)^52^. Recently, a novel approach has been researched; its strategy primarily relies on non-linearization of the harvester's shape. Recently, Shim et al.^54^ proposed a nonlinear piezoelectric harvester using a coupled beam array to overcome these limitations. Their research highlighted the importance of structural nonlinearity and coupling effects from elastic supports, resulting in improved power reliability and wider frequency bandwidth.

The aforementioned reasons motivate us to investigate the effect of nonlinearity in the shape (tapered and straight cross-section area) as well as the fixation method (the number of fastened ends) in order to determine the optimal course of action for obtaining the necessary combination of high output power and wide working frequency. To understand the effect of nonlinearity, it becomes evident when the excitation force subjected to the structure increases—typically when the exciting frequency coincides with the natural frequency of the system. At this point, the structure experiences a large amplitude of vibration, and this amplitude increases for nonlinear structures rather than linear ones.

### Description of the energy harvester's intended designs

**Figure 3:**
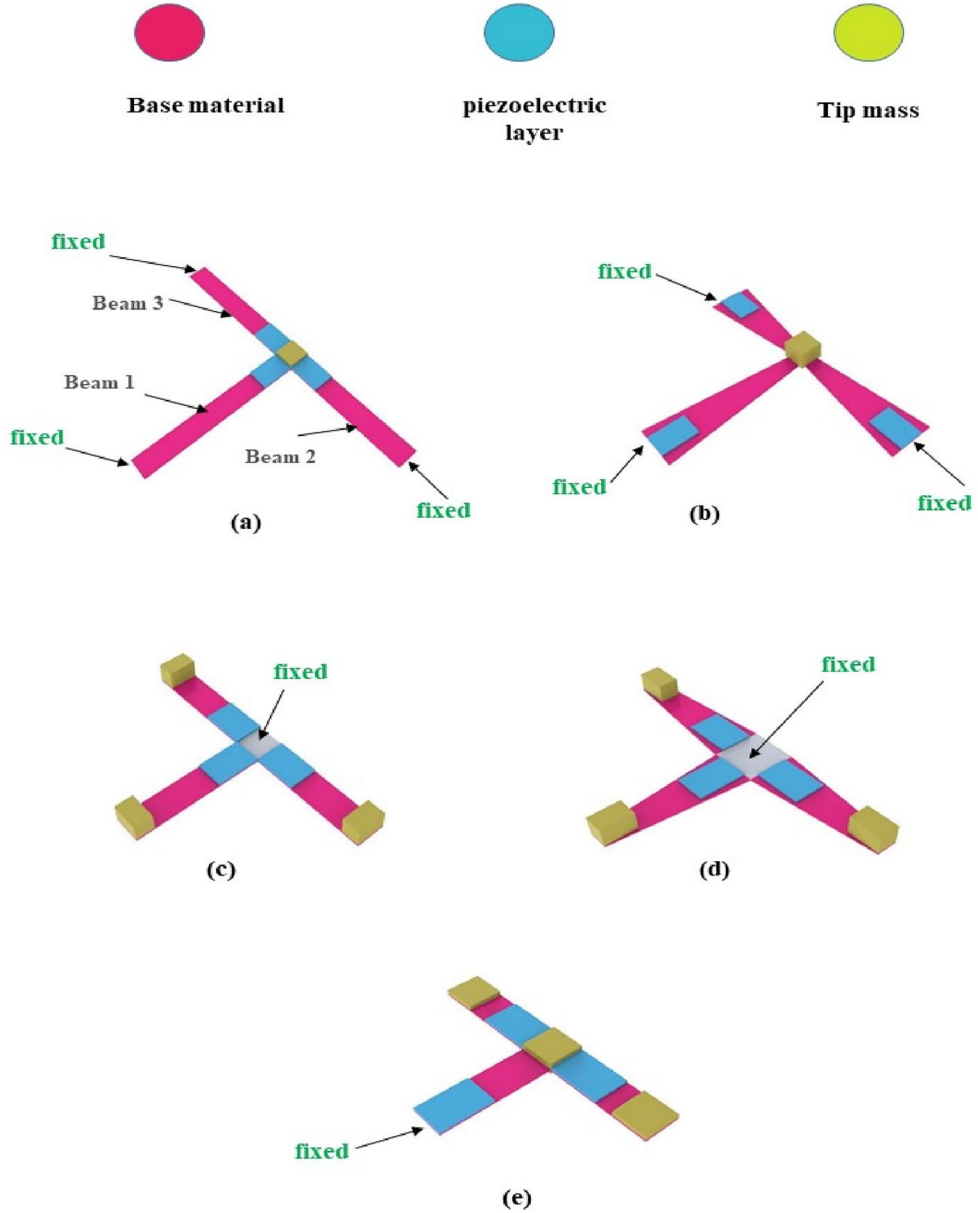
Schematic diagram of the suggested models. (**a**) Three beams fixed from all ends and connected together with a center mass, (**b**) the cross-section area of the beams is varied along their length, (**c**) conventional array of three cantilever carrying tip masses, (**d**) the cross-section area of the cantilevers is varied along their length, (**e**) three beams fixed from one end and the other two ends are free carrying a mass at the center of the harvester.

**Figure 4:**
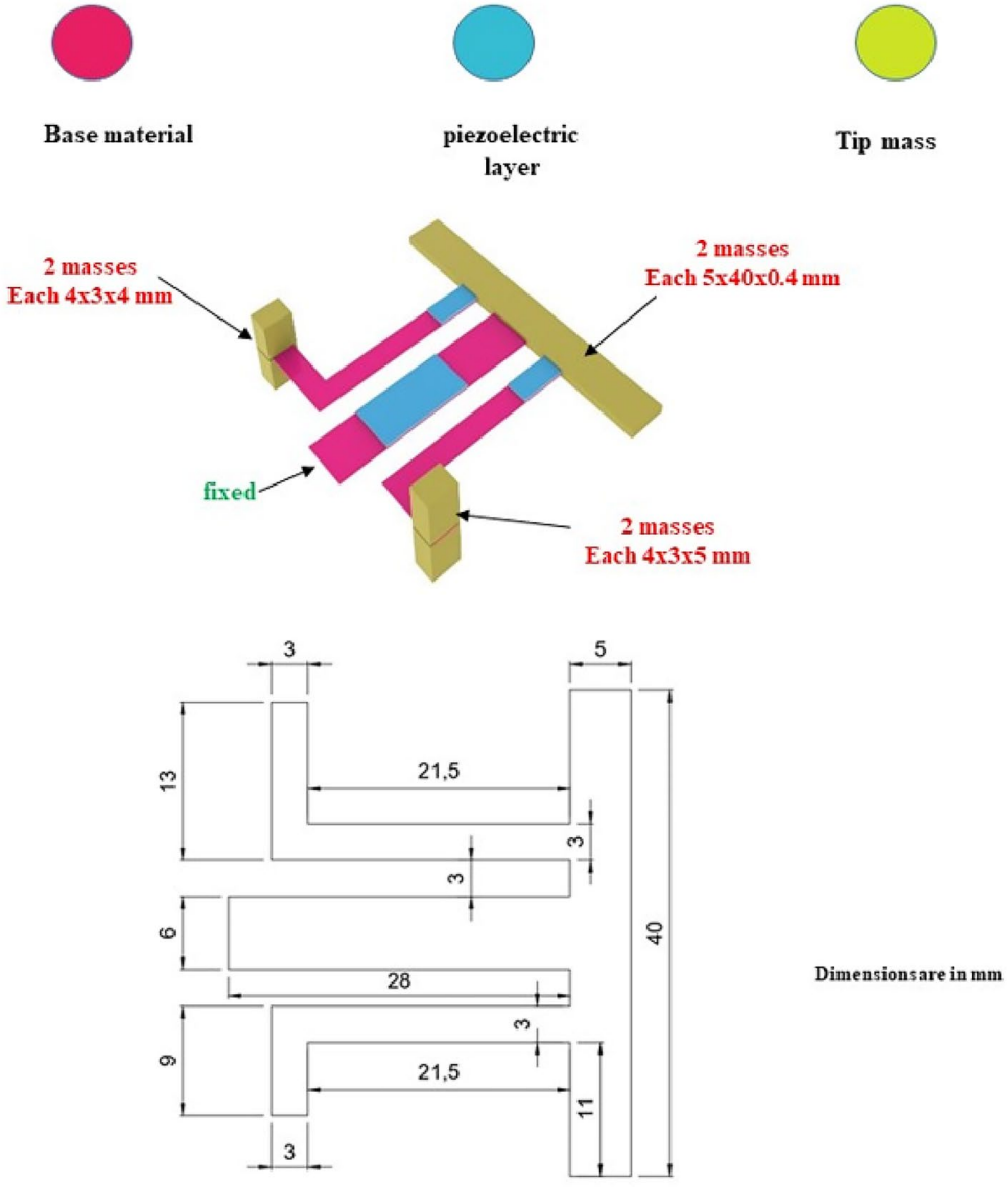
The model from reference^39^ whose dimensions are adjusted as shown to fit the necessary frequency range.

To investigate how nonlinearity influences the harvester's output power, two key parameters are examined here, the first is the effect of changing the cross-section area, and the second is the fixation mechanism. The primary components of the intended designs are three beams connected by three layers of piezoelectric material. Steel serves as the basis material, while Lead Zirconate Titanate (PZT-5H) serves as piezoelectric material. The steel and piezoelectric properties of the components used in the design of piezoelectric energy harvesters are shown in Table 1. The harvester's first design has all the beam ends fixed and the remaining ends joined by a concentrated mass, as seen in Fig. 3a. The impact of tapering the cross-section area is shown in Fig. 3b. The typical array of cantilevers is designed in Fig. 3c; However, it is oriented differently than normal. Also, the effect of changing the cross-section area is considered, as seen in Fig. 3d. Figure 3e shows three beams that are linked and fixed from just one end (the first beam's end). To do a wide-range comparison and assess the efficacy of the planned models, the dimensions of the model from reference^39^ are adjusted as shown in Fig. 4 to fit the necessary frequency range.

**Table 1:** The characteristics of the substrate and piezoelectric materials^55,56^.

Selected material	Steel	Lead zirconate titanate (PZT-5H)
Density (${\text{kg}}/{{\text{m}}}^{3})$	7850	7500
Poisson’s ratio	0.33	0.31
Young’s modulus (GPa)	200	49
Yield strength (MPa)	300	114.8

In each model we design, our goal is to optimize the device's performance by adjusting design variables through an optimization mechanism. Specifically, we aim to align the natural frequencies of the device to achieve a wideband response and maximize output power. Our primary objective throughout these design iterations is to achieve maximum power output from the energy harvester.

## Finite element model and analytical analysis

Both the analytical analysis and the finite element approach (FEA) are presented in this section. FEA is performed using COMSOL 6 Multiphysics for three beams coupled in different directions and utilizing different fixation techniques.

### Analytical analysis

The analytical model for bimorph piezoelectric cantilever is presented in this section, the analytical models of the remaining models will be derived in future work due to complexity. Based on the Euler–Bernoulli beam assumptions, the analytical model of a bimorph cantilever under base excitation is developed^57^. The aim of this analytical approach is to validate the COMSOL model. The piezoelectric harvester's governing equation of motion is written as:

1 $$\frac{{\partial }^{2}M(x,t)}{\partial {x}^{2}}+{c}_{s}I\frac{{\partial }^{5}Q(x,t)}{\partial {x}^{4}\partial t}+{c}_{a}\frac{\partial {Q}_{rel}(x,t)}{\partial t}+{m}_{tot.}\frac{{\partial }^{2}{Q}_{rel}(x,t)}{\partial {t}^{2}}=-\left[{m}_{tot.}+{M}_{tip.}\right]\frac{{\partial }^{2}{Q}_{b}\left(x,t\right)}{\partial {t}^{2}} .$$

where: ${Q}_{rel}(x,t)$ is the beam deflection in the transverse direction with respect to its base at position x and time t, M(x,t) is the moment of internal bending, ${Q}_{b}(x,t)$ is the effective transverse base displacement,${c}_{s}I$ is the composite cross-section's equivalent damping term owing to strain rate damping (where I is the composite cross-section's equivalent area moment of inertia.), ${c}_{a}$ is the damping coefficient for viscous air and ${m}_{tot.}$ is the mass per unit length of the beam, ${M}_{tip.}$ is the tip mass attached at the end of the cantilever. Therefore, the total displacement of the harvester can be expressed as:

2 $${Q}_{tot.}\left(x,t\right)={Q}_{rel}\left(x,t\right)+{Q}_{b}\left(x,t\right).$$

Instead of specifying the damping coefficients in the physical equation of motion, Erturk and Inman^57^ took into account the corresponding undamped equation (by setting ${c}_{s}I$ = ${c}_{a}$= 0 in Eq. (1)) and added modal damping, as is customary, to the equation of motion in modal coordinates.

The harvester mass per unit length ${m}_{tot.}$ can be written as follows:

3 $${m}_{tot.}={d(\rho }_{s}{b}_{s}+2{\rho }_{p}{b}_{p}).$$

where: ${b}_{s}$ is the base material thickness (m), ${b}_{p}$ is the piezoelectric layer thickness (m), and $d$ is the cantilever width, ${\rho }_{s}$ is the density of the base material (${\text{kg}}/{{\text{m}}}^{3}$),${\rho }_{p}$ is the density of the piezoelectric material (${\text{kg}}/{{\text{m}}}^{3}$).

The natural frequency of the ${r}{th}$ vibration mode in short circuit conditions,

${w}_{r}={\uplambda }_{r}^{2}\sqrt{\frac{YI{|}_{eqv.}}{{m}_{tot.}{L}^{4}}}$ can be obtained by solving Eq. (1). The piezoelectric harvester's first mode natural frequency (r = 1) is written as follows:

4 $${w}_{r=1}={\uplambda }_{r=1}^{2}\sqrt{\frac{YI{|}_{eqv.}}{{m}_{tot.}{L}^{4}}} .$$

where: the system's eigenvalues (${\uplambda }_{r}$ for mode r) are derived from:

5 $$\begin{aligned} & 1 +\mathrm{cos\lambda cosh\lambda }+\frac{\uplambda {M}_{tip.}}{{m}_{tot.}L}\left(\mathrm{cos \lambda sinh \lambda }-\mathrm{ sin \lambda cosh \lambda }\right) \\ & \quad -\frac{{\uplambda }^{3}{I}_{t}}{{m}_{tot.}{L}^{3}}(\mathrm{cosh \lambda sin \lambda }\hspace{0.17em}+\hspace{0.17em}\mathrm{sinh \lambda cos \lambda }) \\ & \quad +\frac{{\uplambda }^{4}{{M}_{tip.}I}_{t}}{{{m}_{tot.}}^{2}{L}^{4}}(1 -{ \cos \uplambda \cos {\text{h}} \uplambda }) = 0. \end{aligned}$$

And the following formula is used to get the composite cross-section's equivalent bending stiffness (YI)^40^:

6 $$YI{|}_{eqv.}=\frac{2d}{3}\left[{Y}_{s}\frac{{{b}_{s}}^{3}}{8}+{Y}_{p}\left({\left({b}_{p}+\frac{{b}_{s}}{2}\right)}^{3}-\frac{{{b}_{s}}^{3}}{8}\right)\right].$$

Where: L is the total length of the cantilever,${I}_{t}$ is the tip mass's rotational inertia,${Y}_{s}$ is the elastic modulus of the base material (Pa) and ${Y}_{p}$ is the elastic modulus of the piezoelectric material (Pa).

The mass-normalized eigenfunctions of the related undamped free vibration problem are represented by the eigenfunctions indicated by ${\alpha }_{r}\left(x\right)$^58^:

7 $${\alpha }_{r}\left(x\right)={C}_{r}\left[cos\frac{{\uplambda }_{r}}{L}x-cosh\frac{{\uplambda }_{r}}{L}x+\mu \left(sin\frac{{\uplambda }_{r}}{L}x-sinh\frac{{\uplambda }_{r}}{L}x\right)\right].$$

where $\mu$ is calculated from Eq. (8)^58^:

8 $$\mu =\frac{{\text{sin}}{\uplambda }_{r}-{\text{sinh}}{\uplambda }_{r}+{\uplambda }_{r}\frac{{M}_{tip.}}{{m}_{tot.}L}({\text{cos}}{\uplambda }_{r}-{\text{cosh}}{\uplambda }_{r})}{{\text{cos}}{\uplambda }_{r}+{\text{cosh}}{\uplambda }_{r}-{\uplambda }_{r}\frac{{M}_{tip.}}{{m}_{tot.}L}({\text{sin}}{\uplambda }_{r}-{\text{sinh}}{\uplambda }_{r})}.$$

The following formula expresses the output voltage as a function of frequency $\left(\omega \right)$ due to translational base acceleration^57^:

9 $$v\left(\omega \right)=\frac{j2\omega R{z}_{r}(-{m}_{tot.}{\int }_{0}^{L}{\alpha }_{r}\left(x\right) dx-{M}_{tip.}{\alpha }_{r}\left(L\right))}{\left(2+jwR{C}_{P}\right)\left({\omega }_{r}^{2}-{\omega }^{2}+j2{\upzeta }_{r}{\omega }_{r}\omega \right)+j2\omega R(-{m}_{tot.}{\int }_{0}^{L}{\alpha }_{r}\left(x\right) dx-{M}_{tip.}{\alpha }_{r}\left(L\right)){N}_{r}^{s}} .$$

where: R is the load resistance (Ω),${z}_{r}$ is the term of modal coupling given by Eq. (10)^57^,${C}_{P}$ is the piezoelectric layer's internal capacitance obtained from Eq. (11)^59^, ${\upzeta }_{r}$ is the modal damping ratio and ${N}_{r}^{s}$ is the coupling between the mechanical and electrical systems and is represented by Eq. (12)^59^.

10 $${z}_{r}=-\frac{{e}_{31}\left({b}_{p}+{b}_{s}\right)d}{2} \frac{d{\alpha }_{r}\left(x\right)}{dx}{|}_{x=L}.$$

11 $${C}_{P}=\frac{{\varepsilon }_{33}^{-S} dL}{{b}_{p}}.$$

12 $${N}_{r= {v}_{s}}^{s}\frac{d{\alpha }_{r}\left(x\right)}{dx}{|}_{x=L}.$$

where:${e}_{31}$ is the piezoelectric constant $({e}_{31}=\frac{{d}_{31}}{{s}_{11}^{E}} )$, ${\varepsilon }_{33}^{-S}$ is the component of permittivity and ${v}_{s}$ is the piezoelectric coupling term in the case of a series connection and is given by Eq. (13)^57^.

13 $${v}_{s}=\frac{{e}_{31}d}{2{b}_{p}}\left[\frac{{b}_{s}^{2}}{4}-{\left({b}_{p}+\frac{{b}_{s}}{2}\right)}^{2}\right].$$

Khalili et al.^60^ derived the following expression to calculate the power output $p$ is given as:

14 $$p=\frac{{\left|v\left(\omega \right)\right|}^{2}}{2R}=\frac{R}{{2(R+\frac{1}{2\pi f{C}_{P}})}^{2}}.$$

Where: $f$ is the frequency of vibration in (Hz).

#### Derivation of lumped system analytical model for complex geometries

When dealing with complex geometries, such as the three-dimensional structures considered in our work, deriving analytical models based on continuous systems becomes challenging due to the intricate boundary conditions involved. As a result, we opted to use a lumped system approach, which has been widely employed in the literature for similar complex structures.

**Figure 5:**
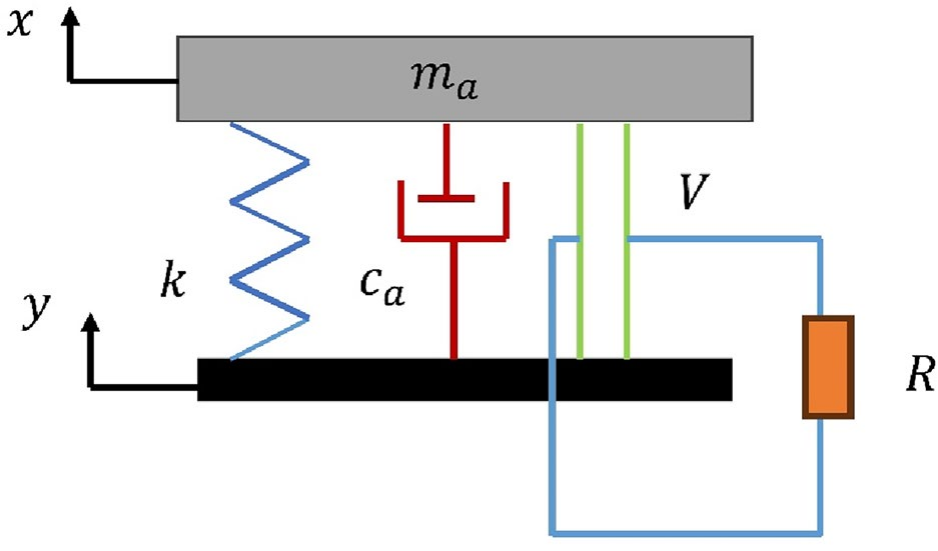
Schematic representation of the lumped system model used for the proposed energy harvester (Model (a)).

To illustrate, we refer to recent studies by Shim et al.^54^ and Qi et al.^61^, where lumped system models were successfully utilized for optimizing piezoelectric energy harvesters with intricate geometries as shown in Fig. 5. Building upon these established methodologies, we derived the governing equations for our proposed energy harvester model (Model (a)).

In our analytical model, we consider the displacement of the base (denoted as y), the mass (${m}_{a}$), and the acceleration of the base (a). Assuming sinusoidal base excitation ($y=Ysin(wt)$), we obtain the governing equation:

15 $${m}_{a}\frac{{d}^{2}x}{d{t}^{2}}+{c}_{a}\left(\frac{dx}{dt}-\frac{dy}{dt}\right)+k\left(x-y\right)=-{m}_{a}\frac{{d}^{2}y}{d{t}^{2}}.$$

Considering the piezoelectric element, with V as the voltage, R as the load resistance, C p ​ as the inner capacitance, and β as the electromechanical coupling coefficient, we incorporate the electromechanical coupling effect into the model:

16 $${m}_{a}\frac{{d}^{2}x}{d{t}^{2}}+{c}_{a}\left(\frac{dx}{dt}-\frac{dy}{dt}\right)+k\left(x-y\right)=-{m}_{a}\frac{{d}^{2}y}{d{t}^{2}}-\beta V.$$

17 $$\beta \left(\frac{dx}{dt}-\frac{dy}{dt}\right)={c}_{p}\frac{dV}{dt}+\frac{V}{R}.$$

#### Finite element modeling of output power

In this section, we present the equation representing the output power used in finite element calculations. This equation, derived from Lefeuvre et al.^62^, models the output power in terms of various parameters such as electrical resistance, base excitation, inertial mass, natural frequency, damping coefficient, and capacitance. The equation undergoes reduction to facilitate computational analysis, resulting in a simplified form.

18 $$Power= \left (\frac{ R {\upbeta }^{2}}{ {\left(\frac{\pi }{2}+R{c}_{p}{\omega }_{n}\right)}^{2}} \frac{{ {{\omega }_{n}}^{4 }{V}^{2}{m}_{a}}^{2}}{{\left({C}_{a}+\left(2R {\upbeta }^{2}/\left(R{c}_{p}{\omega }_{n}+{\left(\frac{\pi }{2}\right)}^{2}\right)\right)\right)}^{2}} \right)$$

After simplification, the output power can be expressed as:

19 $$Power={V}_{op} \left(\frac{2\upbeta }{ \frac{\pi }{2}+R{c}_{p}{\omega }_{n}}+{C}_{a1}\frac{\frac{\pi }{2}+R{c}_{p}{\omega }_{n}}{\upbeta R} \right)$$

Here, β represents the electromechanical coupling coefficient of piezoelectric materials, V is the voltage (V), ${c}_{p}$ is the capacitance (C/V), ${C}_{a}$ is the damping coefficient (Ns/m), ${m}_{a}$ is the dynamic mass (Kg), Y is the base excitation (m), ${\omega }_{n}$ is the natural frequency (Hz), ${{Y\omega }_{n}}^{2}$ is the base excitation (m/s^2^), and R is the electrical load resistance (Ω). This equation serves as the basis for the finite element analysis conducted in our study to determine the output power of the piezoelectric energy harvester under various conditions.

### FEA using COMSOL multi-physics for the models

#### Procedure for the proposed models' optimization

**Figure 6:**
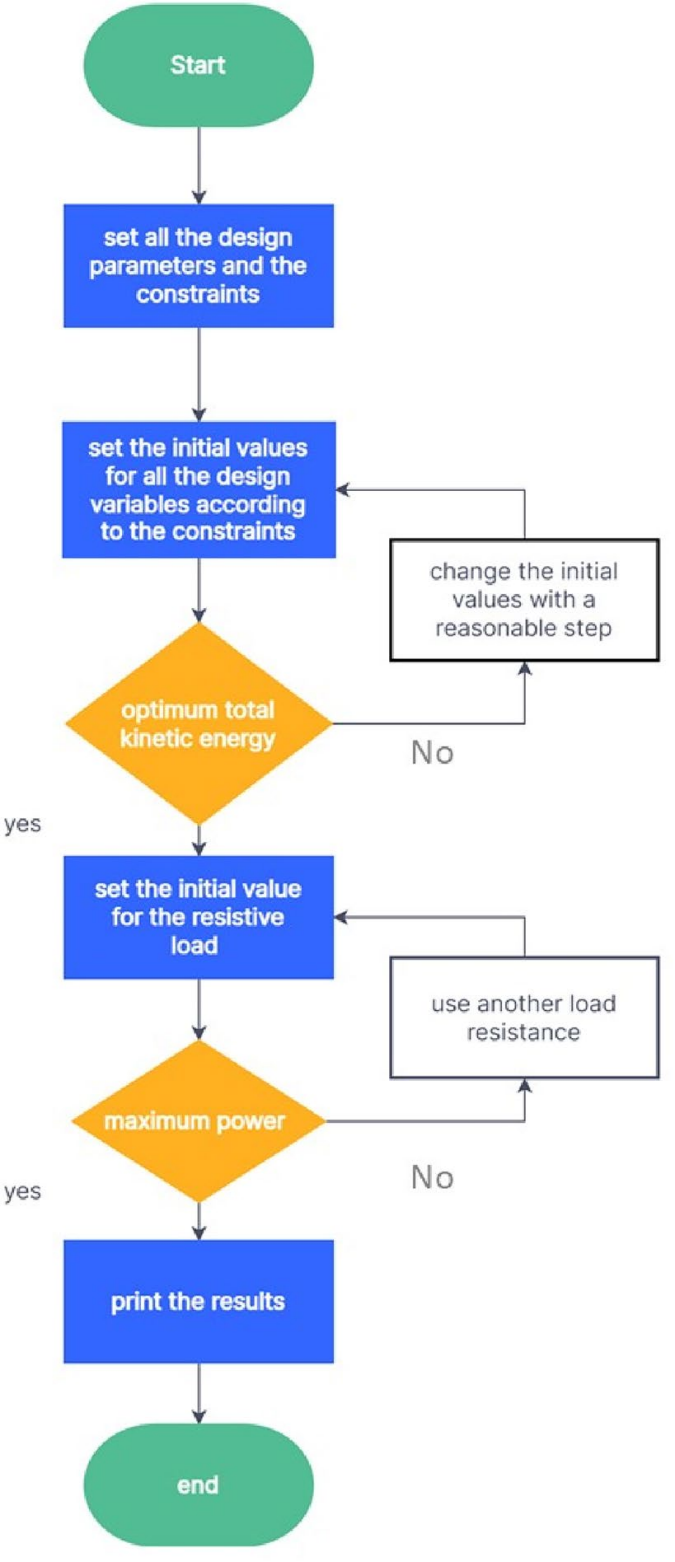
Graphic representation of the optimization process' flow.

In this part, the three beams at each design are optimized to produce the appropriate frequency range. The optimization greatly reduces the number of attempts, efforts, and repetitions. Additionally, optimization increases output power^52,53,63^. The design parameters are the thickness of the base material (t), the length of the first beam ($${L}_{1}$$), the length of the second beam ($${L}_{2}$$), the length of the third beam ($${L}_{3}$$), the width of the first beam ($${w}_{1}$$), the width of the second beam ($${w}_{2}$$), the width of the third beam ($${w}_{3}$$),the height of the first tip mass ($${t}_{1})$$, the height of the second tip mass ($${t}_{2})$$ and the height of the third tip mass ($${t}_{3})$$. For the models shown in Fig. 3b and d, the width of the beams varies throughout their length, with ($${w}_{i1}$$) denoting the widest width of the beam and ($${w}_{i2}$$) denoting the narrowest width of the beam, and (i) denoting the number of the beam. restrictions are imposed here on all of the models' dimensions after specifying all of the design variables to ensure that they are virtually convergent. The optimization module in COMSOL is utilized to optimize the model parameters in order to maximize output power (objective function) and satisfy constraints (necessary frequency range of each beam), upper and Lowest possible value for the design parameters. Thus, the suggested design can be improved. The parameters for the optimization algorithm were carefully chosen to be as follows: the maximum number of iterations was set to 1000, the penalty factor was set to 0.5, its multiplication factor was set to 10, the constraint tolerance was set to 0.01, and the upper bound of the Lagrange multiplier was set to 1.79. The intended application placed restrictions on the harvesters' lower and higher frequency ranges. The chosen application in the present work exploits the vibrations of bridges, with a working frequency range of (2–30 Hz) and a base acceleration of (0.1–0.5 $${\text{m}}/{{\text{s}}}^{2}$$)^64^. The beam width in models (a, c, and e) is 8 mm. Aside from the modified model from reference ^39^, which is depicted in Fig. 4, all of the models' piezoelectric material dimensions (8 × 12 × 0.2 mm) are fixed. The flowchart for the optimization process is shown in Fig. 6. The optimization problem is mathematically formulated by selecting an objective function, design variables, and constraints. The objective function can be a function or a numerical value, such as strain or kinetic energy maximization. Kinetic energy and strain are inversely correlated with output power. The constraints imposed on the dimensions of the harvester, notably the length and width, serve two pivotal purposes. Firstly, they are adjusted to achieve the desired working natural frequency, thereby ensuring optimal performance within the specified frequency range. Secondly, these constraints are designed to protect the harvester against maximum stress levels during operation, thus ensuring structural integrity and reliability.

Therefore, the optimization problem's mathematical formulation is organized as follows:

Objective function: Maximize the total kinetic energy exerted by the harvester.
Inequality constraints: $${H}_{1}: \; 25{\le L}_{1}\le 60 \; \text{mm}$$
  $${H}_{2}: \; 20{\le L}_{2}\le 60 \; \text{mm}$$
  $${H}_{3}: \; 20{\le L}_{3}\le 60 \; \text{mm}$$
  $${H}_{4}: \; 0.1\le t\le 0.3 \; \text{mm}$$
  $${H}_{5}: \; 2{\le t}_{1}\le 5 \; \text{mm}$$
  $${H}_{6}: \; 2{\le t}_{2}\le 5 \; \text{mm}$$
  $${H}_{7}: \; 2{\le t}_{3}\le 5 \; \text{mm}$$
  $${H}_{8}: \; 28{\le f}_{n(\mathrm{1,2},3)}\le 33 \; \text{Hz}$$
  $${H}_{9}: \; \sigma (x,y,z)\le {S}_{y}$$
(For models (b) and (d)): ${H}_{10}: \; 10{\le w}_{11}\le 15 \; \text{mm}$
  $${H}_{11}: \; 4{\le w}_{12}\le 6 \; \text{mm}$$
  $${H}_{12}: \; 9{\le w}_{21}\le 15 \; \text{mm}$$
  $${H}_{13}: \; 4{\le w}_{22}\le 8 \; \text{mm}$$
  $${H}_{14}: \; 10{\le w}_{31}\le 15 \; \text{mm}$$
  $${H}_{15}: \; 4{\le w}_{32}\le 8 \; \text{mm}$$

**Figure 7:**
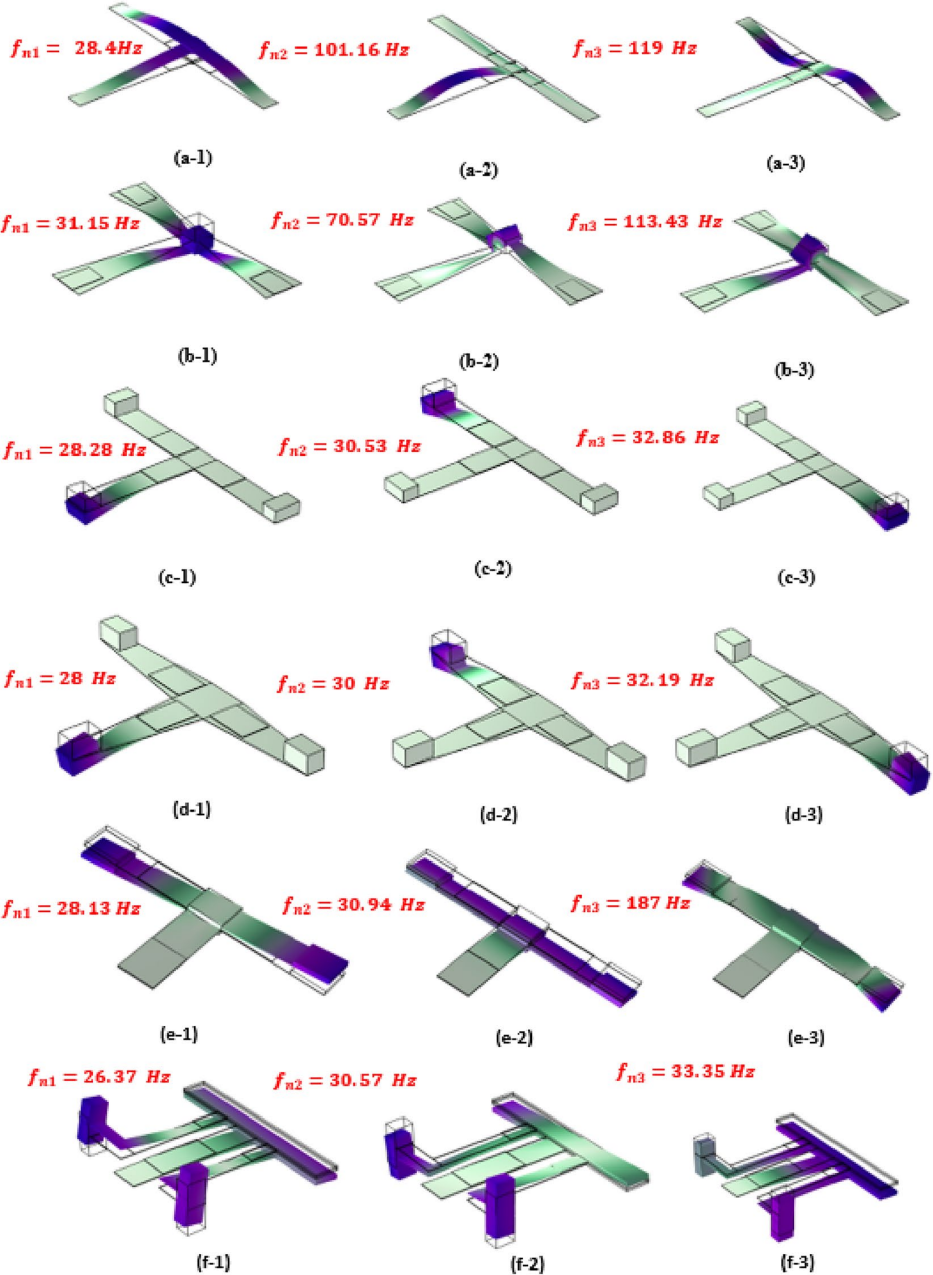
The first three resonant frequencies and the mode shapes of the examined piezoelectric harvesters. (**a**) the mode shapes of model-a (28.4–101.16–119 Hz), (**b**) the mode shapes of model-b (31.15–70.57–113.43 Hz), (**c**) the mode shapes of model-c (28.28–30.53–32.86 Hz), (**d**) the mode shapes of model-d (28–30-32.19 Hz), (**e**) the mode shapes of model-e (28.13–30.94–187 Hz), (**f**) the mode shapes of model-f (26.37–30.57–33.35 Hz).

Using BOBYQA, which stands for bound optimization by quadratic approximation, the results of optimization are obtained. The optimization's lower and upper bounds as well as its outcomes are shown in Table 2. The modal shape of the intended models is depicted in Fig. 7. Table 3 examines the natural frequency of the various models under study. Table 4 investigates the natural frequency and output voltage for various mesh distributions to identify the ideal mesh distribution. This mesh convergence study aims to provide additional consistency and model validation.

**Table 2:** The BOBYQA optimization technique's output results**.**

Dimensions	Lower bound	Upper bound	The optimal results				
			Model a	Model b	Model c	Model d	Model e
${L}_{1}$ [mm]	35	60	60	49.4	35.52	34.74	25
${L}_{2}$ [mm]	30	60	48	40.5	33.25	33	24
${L}_{3}$ [mm]	30	60	53.68	35.3	32.76	33.76	25
$t$ [mm]	0.1	0.3	0.1	0.1	0.1	0.1	0.1
${t}_{1}$ [mm]	2	5	2	5	3.519	5	2.4
${t}_{2}$ [mm]	2	5	–	–	3.566	5	1
${t}_{3}$ [mm]	2	5	–	–	4.42	5	1
${w}_{11}$ [mm]	10	15	–	15	–	11.87	–
${w}_{12}$ [mm]	4	6	–	5	–	5.15	–
${w}_{21}$ [mm]	9	15	–	15	–	12	–
${w}_{22}$ [mm]	4	8	–	5	–	5	–
${w}_{31}$ [mm]	10	15	–	15	–	12.02	–
${w}_{32}$ [mm]	4	8	–	5	–	5.18	–

**Table 3:** The first three natural frequencies (Hz) for the examined models (after optimizing their dimensions).

	${f}_{n1}$ [Hz]	${f}_{n2} [{\text{Hz}}]$	${f}_{n3} [{\text{Hz}}]$
Model a	28.4	101.16	119
*Model* b	31.15	70.57	113.43
Model c	28.28	30.53	32.86
Model d	28	30	32.19
Model e	28.13	30.94	187
Model f	26.37	30.57	33.35

**Table 4:** The mesh convergences study of the natural frequency and maximum voltage for model (a).

Max. element size (mm)	1.2	1	0.8	0.6	0.5	0.4
Number of elements	1351	1840	2980	5796	7872	12,700
${f}_{n1}$(Hz)	28.242	28.202	28.16	28.123	28.106	28.107
Maximum voltage (V)	9.48	9.61	9.52	9.51	9.5	9.5

To identify the ideal mesh distribution, a mesh convergence study was carried out for various mesh distributions.

## FEA COMSOL model validation study

To validate the FEA COMSOL model, two analyses were conducted. In the first, the experimental model described by Erturk and Inman^57^ was replicated in COMSOL with identical geometry and boundary conditions. The results obtained from the FEA were then compared with the experimental data and the outcomes of the analytical approach presented in “Analytical analysis” section. In the second analysis, the output data from model (f) was compared with the experimental data provided in reference^39^.

### Linear model validation

**Figure 8:**
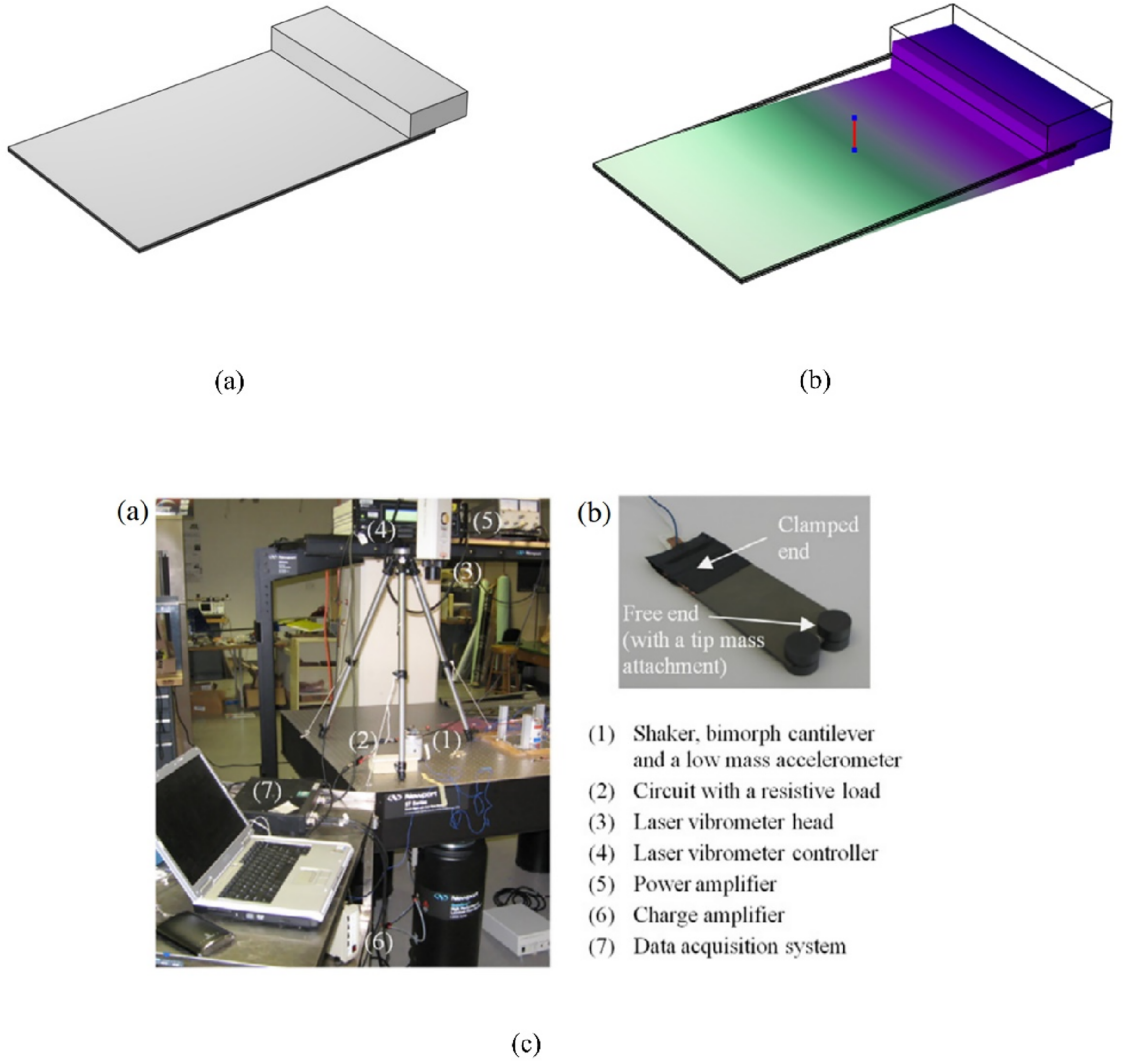
(**a**) Model design and (**b**) mode shapes at the first natural frequency (45.6 Hz), alongside (**c**) experimental setup of the validated model.

**Figure 9:**
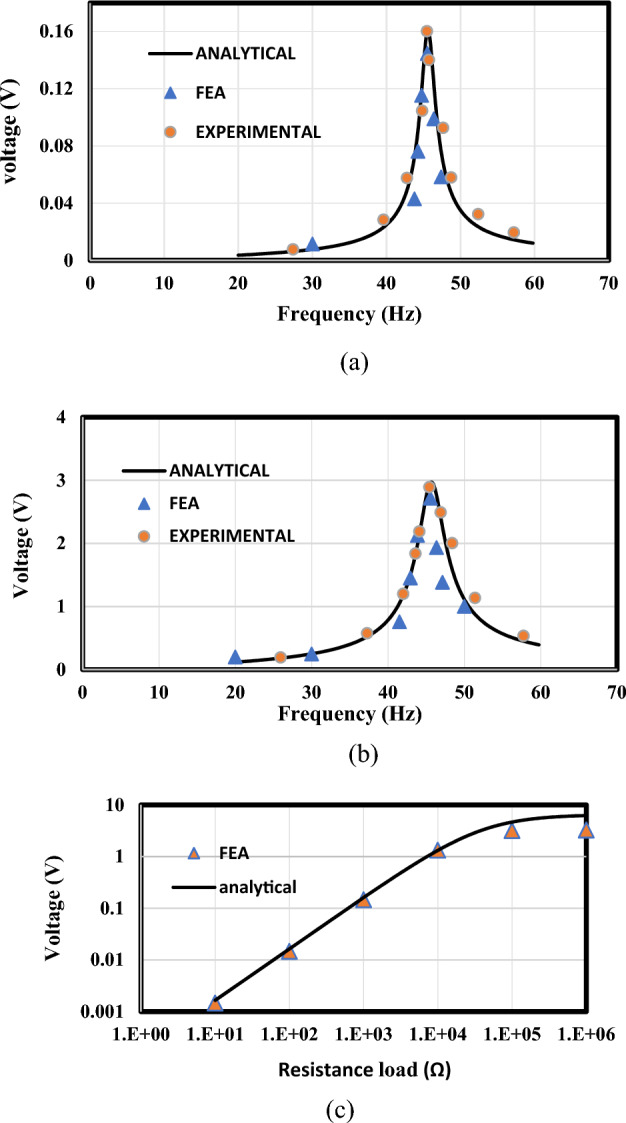
The voltage response of the present FEA findings and the theoretical and experimental findings from ref.^57^. (**a**) Voltage response at R = 1 kΩ, (**b**) voltage response at R = 33 kΩ, (**c**) peak voltage amplitudes at various levels of load resistance.

A bimorph cantilever arrangement with series connections of piezoceramic layers was introduced by Erturk and Inman. The arrangement has a 12 g tip mass attached to the cantilever. Table 5 summarizes the substructure and piezoceramic layers' geometric and material characteristics. Figure 8 illustrates both the model design and the experimental setup of the validated model, along with the mode shapes at first natural frequency (45.6 Hz). The piezoelectric beam's natural frequency and mode shape are investigated using FEA. The frequency evaluated using FEA is 45.661 Hz compared to the results of reference^57^ which was 45.6 Hz. The findings of our FEA COMSOL model, the experimental results, and the analytical results are in good agreement, as shown in Fig. 9.

**Table 5:** The bimorph cantilever's geometric and material parameters were employed for the experimental and analytical validation of the FEA model^57^.

Geometric specifications	Piezoelectric material	Base material	Material specifications	Pzt-5A (piezo.)	Brass (base material)
Length, $L [{\text{mm}}]$	50.8	50.8	Density, $\rho$ [${\text{kg}}/{{\text{m}}}^{3}]$	7800	9000
Width, d [mm]	31.8	31.8	Young’s modulus of elasticity, Y [G Pa]	66	105
Thickness, b [mm]	0.26 (each)	0.14	piezoelectric constant, ${d}_{31}$ [pm ${{\text{V}}}^{-1}]$	− 190	–
Tip mass, ${M}_{tip.}$ [kg]	-	0.012	Permittivity ,${\varepsilon }_{33}^{-S}$ [F ${{\text{m}}}^{-1}]$	1.328 × ${10}^{-8}$	–

### Complex model validation

**Figure 10:**
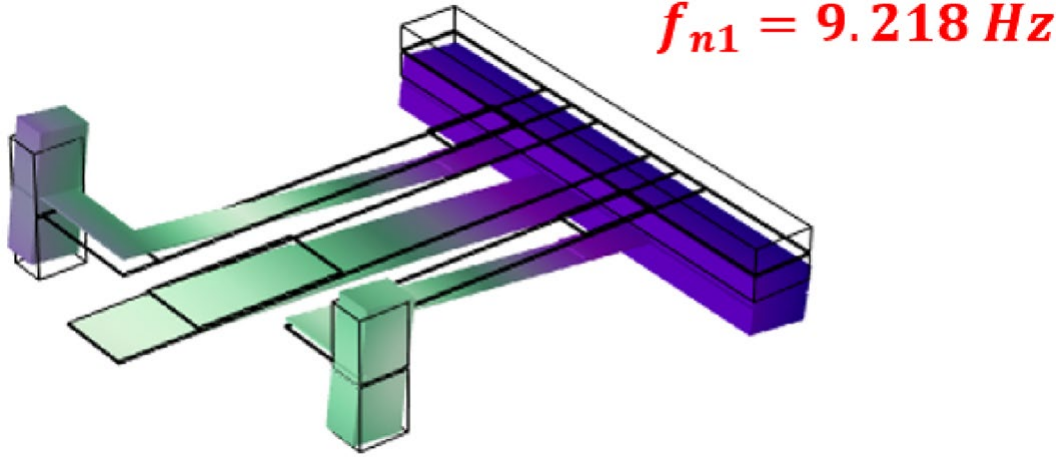
The natural frequency and the first mode shape of the harvester that is used to validate the FEA model.

**Figure 11:**
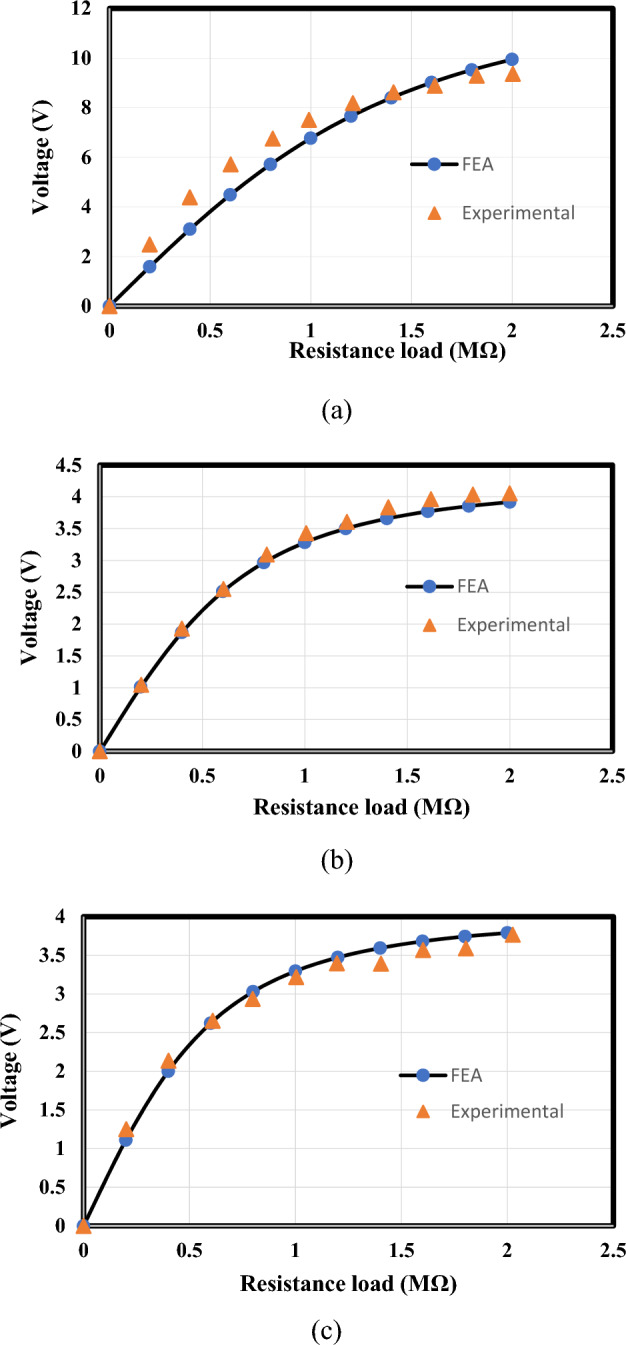
Peak voltage amplitudes at various levels of load resistance of the present FEA findings and the experimental findings from ref.^39^. (**a**) At ${f}_{n1}$=9.21 Hz, (**b**) at ${f}_{n1}$=15.78 Hz, (**c**) at ${f}_{n1}$=19.43 Hz.

This section compares the FEA results of model (f) with the experimental results of^39^. The material and geometric parameters of the harvester used for experimental validation are shown in Table 6. The piezoelectric harvester's first natural frequency and mode shape, estimated by the FEA, are shown in Fig. 10. The peak voltage amplitudes of the FEM findings and the experimental results of reference^39^ are shown in Fig. 11 at different load resistance values. Good convergence is shown in the results, giving confidence to use FEM in this investigation.

**Table 6.** The material and geometric parameters of the harvester used for experimental validation of model (f)^39^.

Geometric specifications	Value	Material specifications	Value
Length of the middle beam, [mm]	41	piezoelectric constant, ${d}_{31}$[pm ${{\text{V}}}^{-1}]$	274
Length of the right-wing beam, [mm]	29.5	Young’s modulus of elasticity of PZT unimorph, [GPa]	60
Length of the left-wing beam, [mm]	35	Young’s modulus of elasticity of brass, [GPa]	110
The tip mass of the middle beam, [mm]	5 × 40 × 2	The dielectric constant of PZT unimorph,${\varepsilon }_{33}$	3400
The tip mass of wing beams, [mm]	3 × 4 × 5	Density of brass, $\rho$ [${\text{kg}}/{{\text{m}}}^{3}]$	8960
Dimensions of the piezoelectric element located at the main beam, [mm]	12 × 6 × 0.2		
Dimensions of the piezoelectric element located at the wing beams, [mm]	6 × 3 × 0.2

## Results and discussion

**Figure 12:**
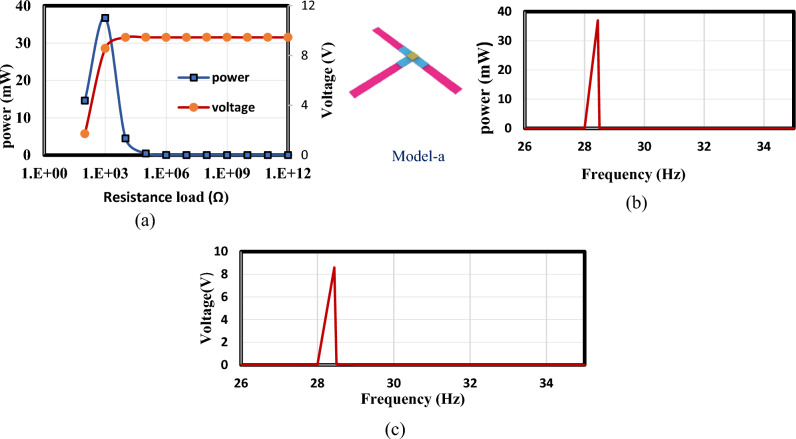
(**a**) Optimization of model-a's load resistance at 28.4 Hz frequency, (**b**) frequency response of the power at 1 kΩ, (**c**) frequency response of the voltage at 1 kΩ. The output power and frequency bandwidth are both impacted by load resistance optimization.

**Figure 13:**
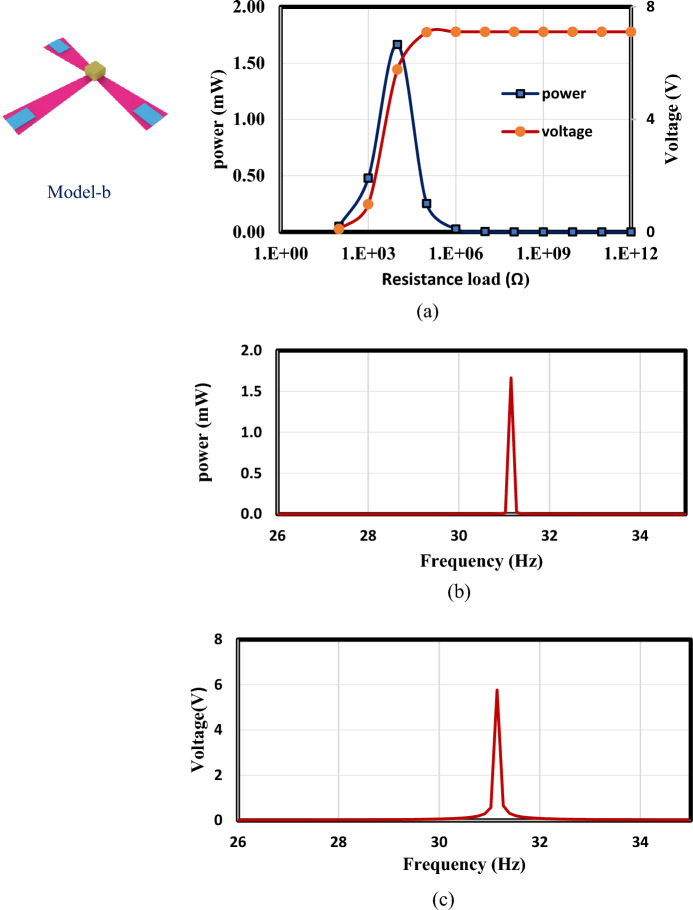
(**a**) Optimization of model-b's load resistance at 31.15 Hz frequency, (**b**) frequency response of the power at ${10}^{4}$ Ω, (**c**) frequency response of the voltage at ${10}^{4}$ Ω.

**Figure 14:**
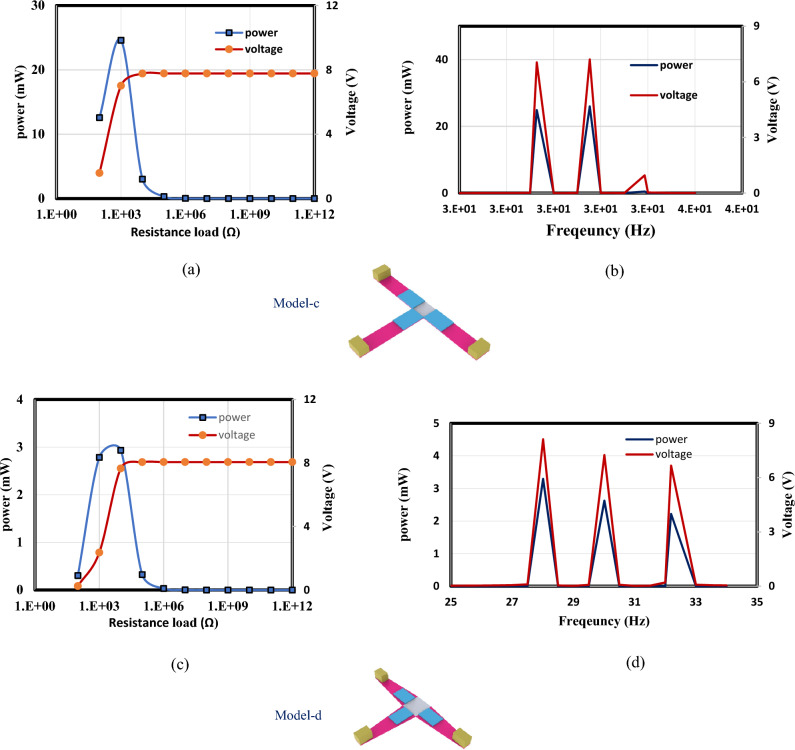
(**a**) Optimization of model-c's load resistance at 28.28 Hz frequency, (**b**) frequency response of the power and the voltage at ${10}^{3}$ Ω, (**c**) the load resistance of model-d is optimized at a frequency of 28 Hz, (**d**) frequency response of the voltage and power at ${10}^{4}$ Ω.

**Figure 15:**
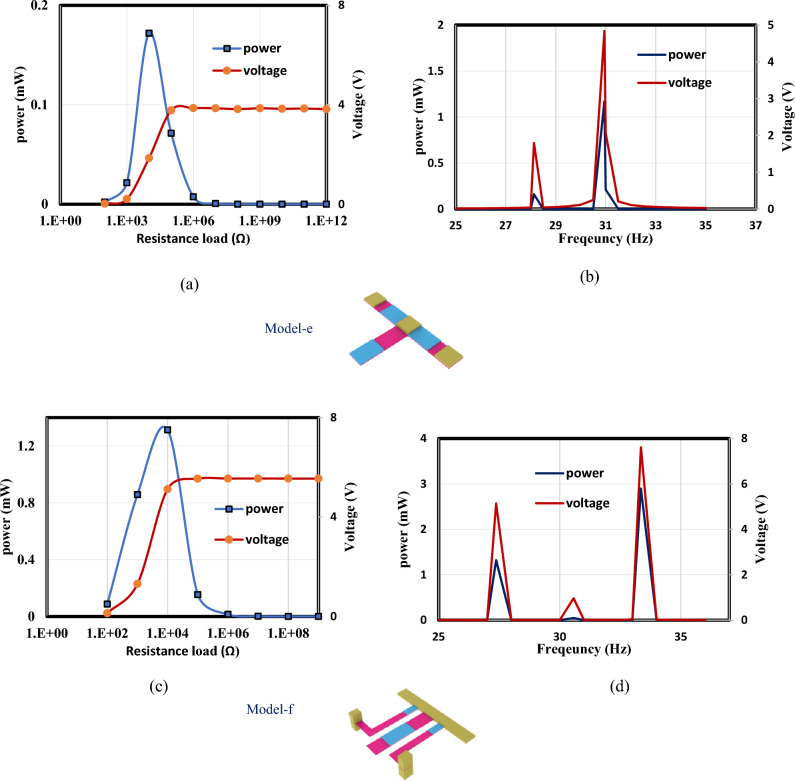
(**a**) Load resistance of model-e is optimized at 30.94 Hz frequency, (**b**) frequency response of the power and the voltage at ${10}^{4}$ Ω, (**c**) The load resistance of model-f is optimized at a frequency of 27.37 Hz, (**d**) frequency response of the voltage and power at ${10}^{4}$ Ω.

In this section, the optimized models in Figs. 3 and 4 are simulated using FEA to investigate the effect of load resistance. Based on an applied frequency range from 25 to 40 Hz, a base excitation acceleration of 0.5 ${\text{m}}/{{\text{s}}}^{2}$, and damping coefficient of 0.002 the output voltage and output power are evaluated. In order to generate the output power, one side of the piezoelectric layer is chosen to serve as the ground and attached the other (the terminal side) to the electrical load resistance. For the lowest frequency (the first natural frequency), the resistance dependence of electrical power is investigated for all models. The optimal resistance of model (a) is 1 kΩ, as shown in Fig. 12a. The harvester's highest power at a frequency of 28.4 Hz is 37 mW; Fig. 12b and c demonstrate the output power's frequency response and the voltage at a resistance load of 1 kΩ. According to Fig. 13a, it is found that the optimal resistance of model (b) is ${10}^{4}$ Ω. At a frequency of 31.15 Hz, the harvester's maximum power is 1.67 mW; Fig. 13b and c show the output power's frequency response and the voltage at a ${10}^{4}$ Ω resistance load, respectively. The optimum resistance of model (c) is 1 kΩ as displayed in Fig. 14a. The harvester's maximum power is 24.59 mW at a frequency of 28.28Hz; Fig. 14b demonstrates the output power's frequency response and the voltage at a 1 kΩ resistance load. Based on Fig. 14c, model (d)'s optimal resistance is ${10}^{4}$ Ω. At a frequency of 28 Hz, the harvester's maximum power is 3.29 mW; Fig. 14d shows the output power's frequency response and the voltage at a ${10}^{4}$ Ω resistance load. As illustrated in Fig. 15a, the optimum resistance for model (e) is ${10}^{4}$ Ω. At a frequency of 30.94 Hz, the harvester's maximum power is 1.17mW; Fig. 15b shows the output power's frequency response and the voltage at ${10}^{4}$ Ω as resistance load. Model (f)'s optimal resistance is ${10}^{4}$ Ω, as shown in Fig. 15c. Figure 15d shows the output power's frequency response and the voltage at a ${10}^{4}$ Ω resistance load, respectively. The harvester's highest power is 1.32 mW at a frequency of 27.37 Hz.

**Figure 16:**
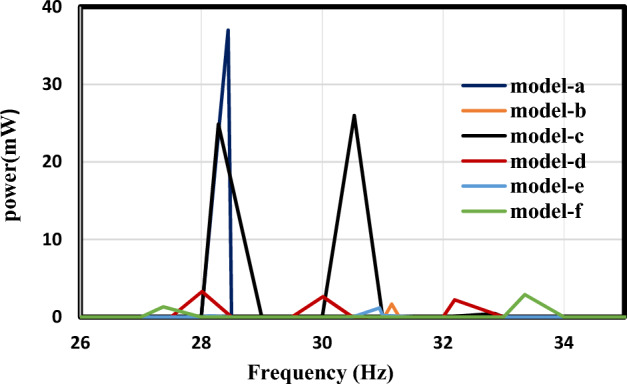
Comparison of the frequency response and power output for the investigated models.

According to the previous results, model (a) generates the highest output power out of all the designs, followed by the conventional array of cantilevers. To thoroughly analyze the results and ensure the validity of the design, power per unit volume is evaluated for both the piezo-material and the base material. This evaluation decides the most suitable nonlinear design. Figure 16 compares the output power for the models under investigation. The detailed comparison between the various models is described in Table 7. This research demonstrates that Model (a) has the optimal nonlinear design with power per unit volume of the substrate and piezo-material as follows **(**$\frac{{P}_{max}}{{volume}_{piezo. material}}=0.64 \; \mathrm{ mW }/{{\text{mm}}}^{3} \; \mathrm{and} \; \frac{{P}_{max}}{{volume}_{substrate}}=0.1436\; \mathrm{ mW }/{{\text{mm}}}^{3}$). And to further enhance the comparison Table 8 compares the merits and drawbacks of the current proposed designs. The advantages of model (a) make it the preferred design, and its drawbacks can be mitigated by utilizing an array due to its simple design.

**Table 7:** Comparison of the different models' predictions including the optimal resistive load, maximum power output, power generated by the piezoelectric material and substrate per unit volume, as well as the max. stress and factor of safety of the substrate and the piezoelectric material.

Model	Optimum resistance [Ω]	Maximum power [mW]	$\frac{{P}_{max}}{{volume}_{piezo. material}}$ [mW/${{\text{mm}}}^{3}]$	$\frac{{P}_{max}}{{volume}_{substrate}}$ [mW/${{\text{mm}}}^{3}]$	Substrate max. stress [MPa]	Piezo max. stress [MPa]	F.O.S (substrate)	F.O.S (piezo material)
a	${10}^{3}$	**37**	**0.64**	**0.1436**	**200**	**24.8**	**1.5**	**4.62**
b	${10}^{4}$	1.67	0.028	0.00375	144	8.52	2.083	13.49
c	${10}^{3}$	24.59	0.426	0.045	210	20.5	1.429	5.6
d	${10}^{4}$	3.29	0.057	0.0047	74	8.9	4.054	12.89
e	${10}^{4}$	1.17	0.02	0.0034	13	3.8	23.077	30.12
f	${10}^{4}$	1.32	0.061	0.0272	30	8	10	14.35

Significant values are in bold.

**Table 8:** Comparison between the advantages and the disadvantages of the current proposed designs.

Model	Advantages	Limitations
a	Highest power output Ability to easily create an array Simple configuration	Limited bandwidth
b	Tapered beams improve stress distribution	Lower power density compared to other models Complexity in fabrication Limited bandwidth
c	Simplicity in design Good bandwidth	Lower power density compared to model (a) Stress concentration near the fixed ends
d	Good bandwidth Tapered beams improve stress distribution	Lower power density compared to other models Complexity in fabrication
e	Simplicity in design Moderate bandwidth	Lower power density compared to other models
f	Good bandwidth	Lowest power output Complex design

This comparison also demonstrates that changing the harvester's cross-section area has no beneficial impact on the harvester's efficiency. Instead, the dimensions should be carefully considered to prevent potential failure due to bending stress because of the changed cross-section area.

The mode shape plays a pivotal role in understanding the behavior of the energy harvester's individual components. Beginning with model (a), at any of its natural frequencies, all three beams exhibit significant deflection, with maximum deflection and stress concentrated near the proof mass. This configuration enables efficient energy harvesting from all three piezoelectric patches. Conversely, in model (c), a detailed examination of the mode shapes reveals a contrasting scenario. Each natural frequency prompts extensive movement in one of the three beams, while the remaining beams remain relatively static. Understanding how the parts move, called mode shapes, is important. It helps us see how energy moves around in the device and how well it works. By studying these mode shapes, we can figure out how to make the energy harvester work even better. This is a key step in improving its performance and efficiency.

### Comprehensive analysis of model (a)

**Figure 17:**
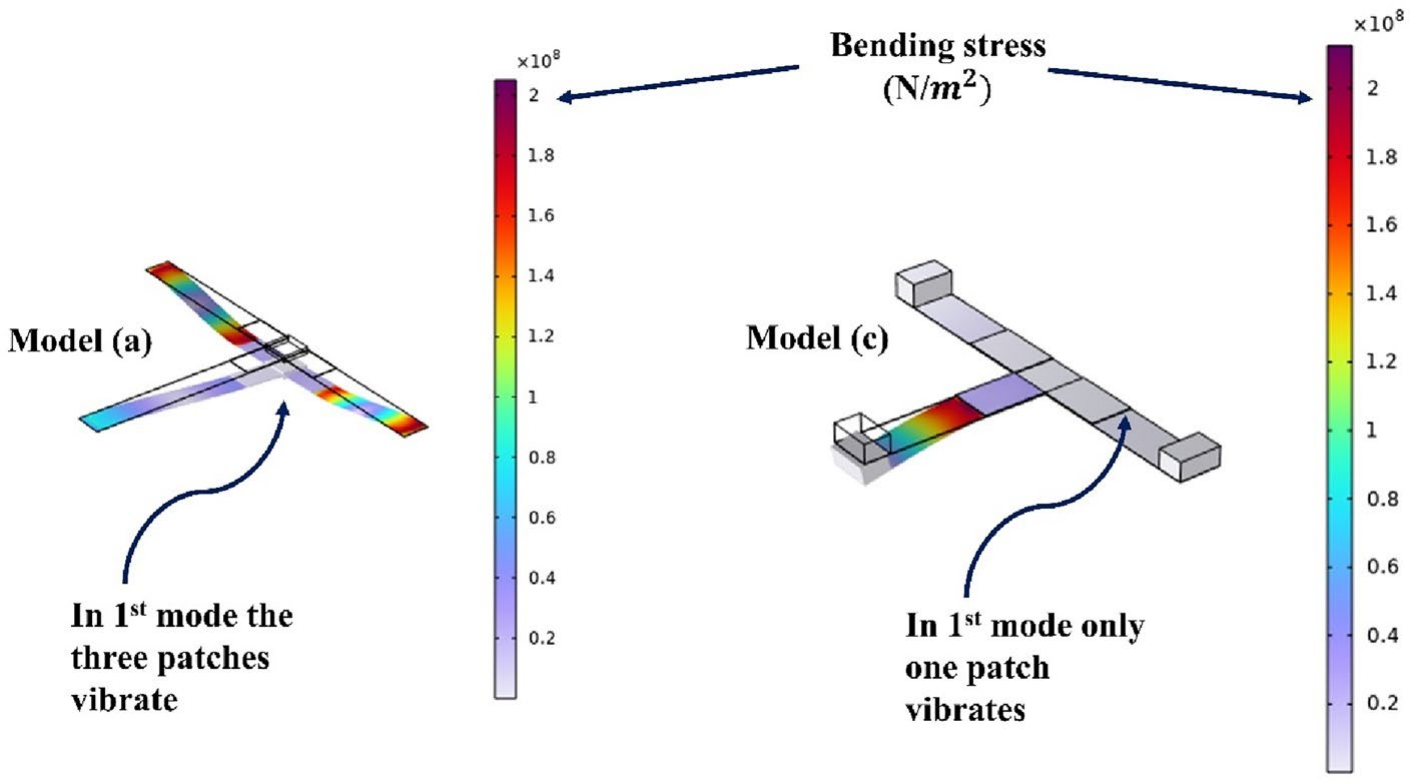
Comparison between the total stress of models (**a**) and (**c**).

To further illustrate why model (a) showed the best efficiency out of all the investigated models, it is essential to mention that electrical displacement and electric field are proportional to the mechanical strain, and mechanical stress. The total bending stress can be derived from this equation ${\upsigma }_{bending}=\frac{{{\text{M}}}_{max}{{\text{y}}}_{max}}{I}$ where ${{\text{y}}}_{max}$ is the distance from the neutral axis, I is the second is the moment of area and ${{\text{M}}}_{max}$ is The maximum bending moment. The bending stress is caused by the mass effect, inertia, and external force due to the base excitation. Although the bending stress in model (a) isn’t higher than in model (c) as mentioned in Fig. 17 in model (a) the three patches attached to the harvester can be utilized in contrast to model (c) where the output power can be obtained from one patch only in every single mode.

**Figure 18:**
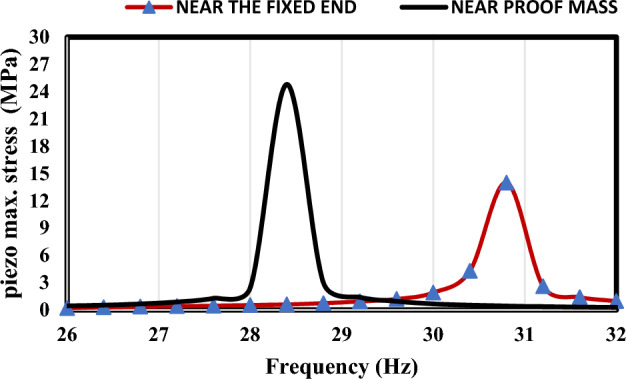
Stress distribution analysis of model (a) with piezoelectric layer positioned near tip mass compared to near the fixed end.

Figure 18 illustrates that by positioning the piezoelectric layer near the tip mass, stress distribution is maximized across the device, thereby enhancing the output power generated by the piezo material.

#### Reliability study of Model (a)

**Figure 19:**
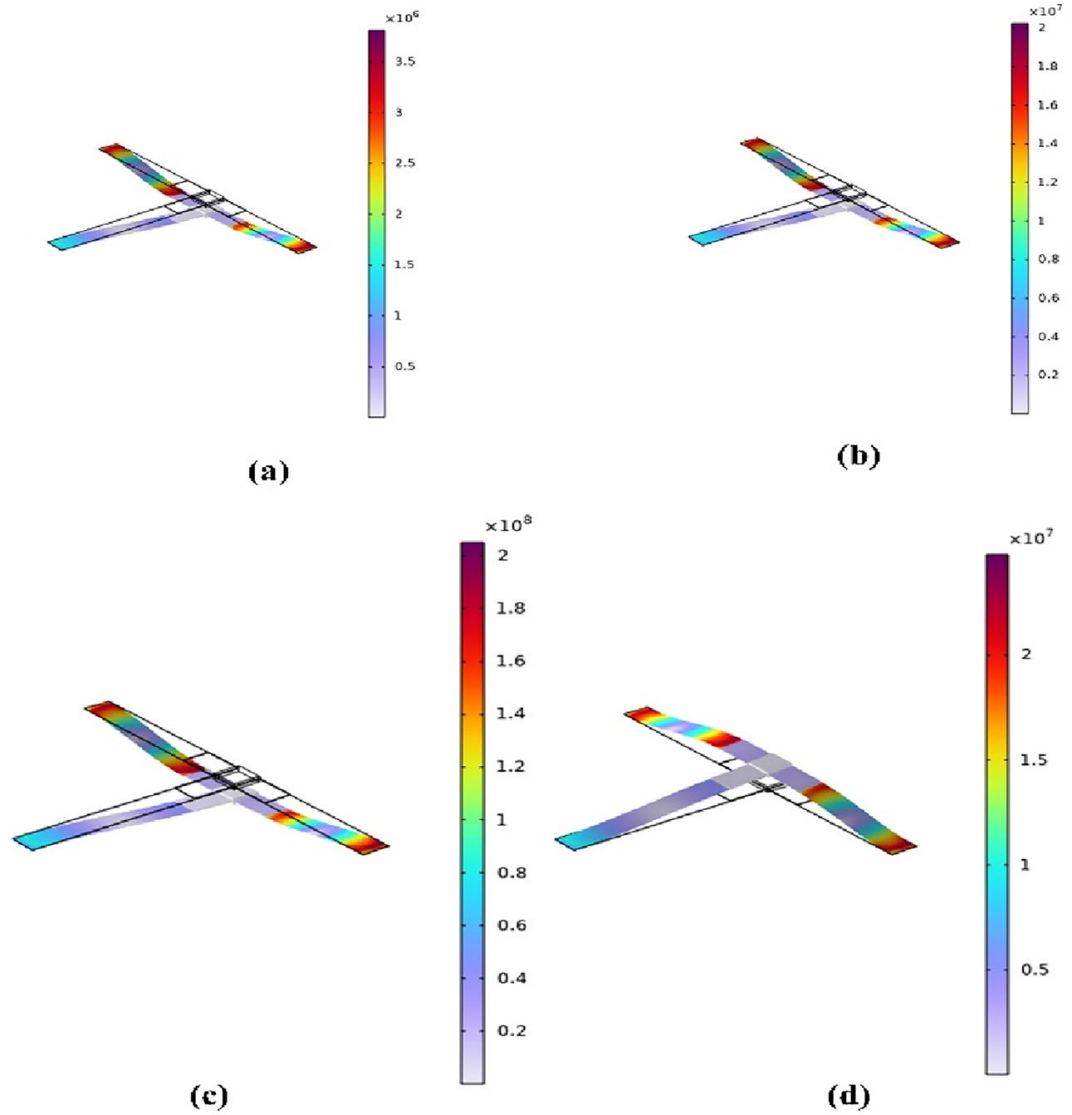
Stress distribution analysis of the model under different frequencies: (**a**) at 26 Hz, (**b**) at 28 Hz, (**c**) at the first natural frequency 28.4 Hz, and (**d**) at 28.8 Hz.

In this section, we present an analysis of the stress distribution within the model under different frequencies. Figure 19 illustrates the stress distribution at various frequencies: (a) at 26 Hz, (b) at 28 Hz, (c) at the first natural frequency 28.4 Hz, and (d) at 28.8 Hz. The results indicate that the maximum stress distribution occurs when the frequency of the application coincides with the natural frequency of the device. Specifically, the maximum bending stress at the base material is 200 MPa, while the maximum bending stress in the piezo material is 24.8 MPa. It's noteworthy that these stress values are below the yield strength of the materials used, ensuring the reliability of the design.

Since it has previously been shown that Model (a) is the most successful nonlinear design, it is crucial to carefully consider its design parameters and how they affect the natural frequency to select the appropriate dimensions for each application. This is so because the operating frequency for each application varies.

**Figure 20:**
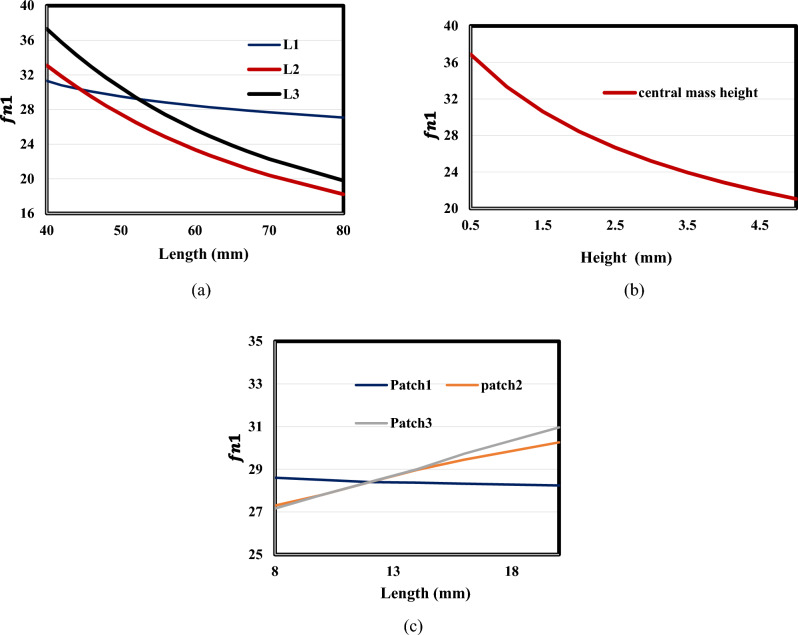
Effect of beam characteristics on resonance frequency (beam length, central mass height, and PZT length), (**a**) length (**b**) central mass height (**c**) path length.

The parametric impact has been investigated in this section. The effect of changing the first, second, and third beam lengths to natural frequency is shown in Fig. 20a. It is evident that L_1_ has a negligible impact on the first natural frequency (${{\varvec{f}}}_{{\varvec{n}}1}$**)** while L_2_ and L_3_ have nearly identical effects. Figure 20b shows how lowering the natural frequency is caused by raising the central mass. Figure 20c demonstrates a positive correlation between the patch length and the natural frequency. As the length of the patch increases, the natural frequency also increases.

#### Fabrication study

In this study, we have conducted a thorough investigation into the fabrication considerations for the piezoelectric energy harvester (PEH) designs. Our focus was on ensuring that the chosen design not only met performance objectives but also facilitated practical fabrication processes. To this end, we opted for model (a) due to its simplicity and ease of fabrication. This model features three beams joined together from one end by a tip mass, with the other ends fixed, simplifying the fabrication process compared to more complex designs found in the literature^19,24,44,46,51^. By deliberately selecting a simpler design, we aimed to streamline the fabrication process and minimize potential errors. Furthermore, the use of standard manufacturing techniques and materials ensures feasibility in fabrication processes. Notably, the fabrication process for model (a) can be efficiently performed using computerized CNC machines, enhancing the reproducibility and accuracy of fabricated device components. CNC machines can cut steel sheets to the desired dimensions. CNC machines are indeed capable of achieving tolerances as low as 0.003 mm to 0.01 mm^65–67^, ensuring precise fabrication according to the specified dimensions. Therefore, based on the availability of suitable materials and the capabilities of CNC machining, we are confident in the feasibility of fabricating the dimensions outlined in Table 2. Piezoelectric fabrication, applications, design and fabrication of complex geometry, and an array of beams were listed in the literature^68–79^.

A mechanical shaker, capable of supplying a controlled force across a wide frequency range, is employed to excite the vibration of the harvester. The shaker is driven at various voltages and frequencies by a function generator and a power amplifier to deliver a sinusoidal force of the desired magnitude. An accelerometer is utilized to monitor the vibration acceleration, while a signal conditioner amplifies the acceleration signal. Additionally, the output voltage of the prototype is monitored using a Digital Oscilloscope. This setup allows for precise measurement and analysis of the electrical characteristics of the constructed module.

Furthermore, it's important to note that the output voltage from the harvester is in AC form, which requires conversion into DC for practical applications. This conversion can be achieved using a rectifier circuit. Additionally, to store the harvested energy for later use, supercapacitors can be employed to act as energy storage devices. This enables the harvested power to be saved and utilized later, enhancing the overall efficiency and versatility of the energy harvesting system.

Model (a) is the most effective model that produces maximum output power among all models, but it falls short in terms of broadband performance throughout a variety of potential working frequencies. An array of three compound beams has been built to address this issue and to develop an integrated harvester that can generate high output power over a wide range of working frequencies.

### Analysis of resultant structure

**Figure 21:**
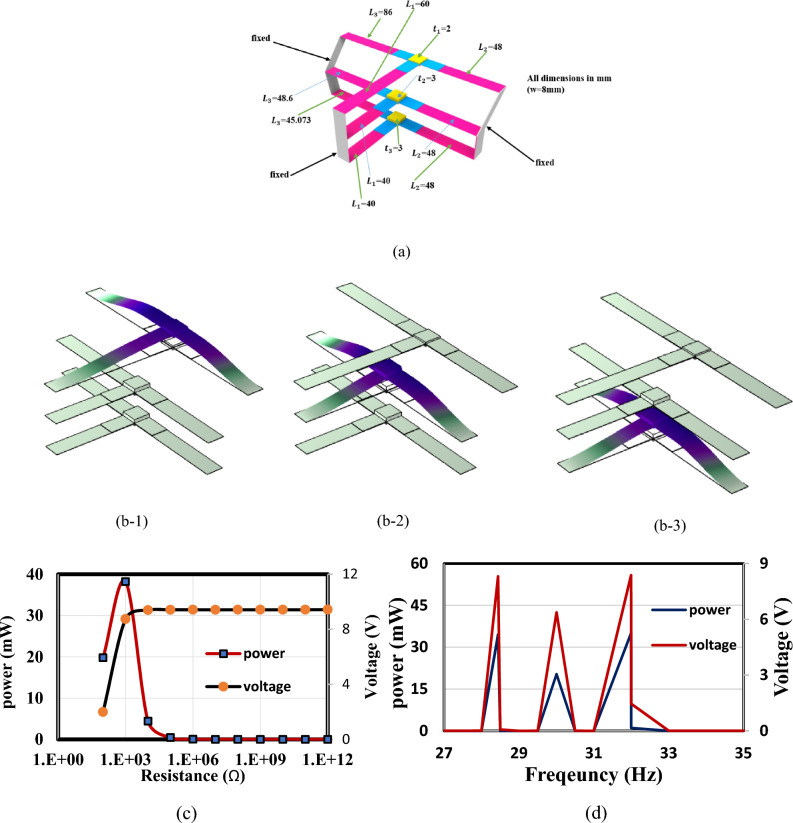
Comprehensive Analysis of the resultant structure. (**a**) Construction of the resultant array of model (a) with optimized natural frequencies. (**b**) Mode shapes corresponding to the first three natural frequencies. (**c**) Investigation of electrical power dependence on resistance, revealing an optimal resistance value of 10^3^ ohms. (**d**) Frequency response of the harvester, illustrating the highest power output at various frequencies.

Figure 19 illustrates various aspects of the analysis. Figure 21a describes the construction of the resultant array of model (a), optimized to exhibit natural frequencies at 28.44 Hz, 30 Hz, and 32 Hz. Figure 21b depicts the mode shapes corresponding to the first three natural frequencies. Furthermore, Fig. 21c investigates the electrical power dependence on resistance, revealing an optimal resistance value of 10^3^ ohms. Finally, Fig. 21d presents the frequency response of the harvester, showcasing the highest power output of 34.5 mW at a frequency of 28.44 Hz, 20 mW at 30 Hz, and 35.1 mW at 32 Hz. By deploying multiple arrays along the bridge, we expect covering a larger bandwidth, ensuring alignment with the operational frequencies commonly encountered in real-world settings.

## Conclusion

In this paper many models were investigated to determine the optimal design in terms of broadband natural frequency and maximum output. Utilizing the optimization COMSOL module, the frequency range and dimensions of the harvester were optimized to guarantee broad band natural frequency and maximum output power. The findings of the comparison between the FEA model's results and those of the analytical and experimental results revealed a high degree of agreement, which validates our model. The conclusion is listed in here the following points:

- For the PEH, there are two potential nonlinear designs. The first one attempts to have a broadband width that will allow it to operate over a large frequency range. The models (e) and (f) serve as examples of this design. However, this design has a flaw in that it only produces a few microwatts even at resonance frequency, which is too little to power a tiny sensor that uses milliwatts of electricity. The second design offers a high intensity of power at the resonance frequency, but in contrast to the first design, it is unable to accommodate a broad variety of working frequencies. Controlling the motion restriction allows for this design. To achieve our objective in this situation, we modified the fixation's endpoints. These designs are demonstrated by models (a) and (b).
- After a thorough analysis of the influence of changing the cross-section area on the power produced by the harvester, it is evident that changing has no beneficial effect on the output power.
- Reliability analysis revealed that models e and f offer increased safety and reliability, making them suitable for environments with higher acceleration. This emphasizes the importance of reliability in optimizing piezoelectric energy harvesters for various applications.
- Model (a) was selected as the most properly suited nonlinear design after a thorough analysis and comparison of the investigated models. The simplicity of the design and the high output power (37 mW) in comparison to the other models are two main factors in selecting this design.
- In order to operate and produce high output power over a broad operating frequency range, an array of three compound beams has been constructed. The ideal resistive load was ${10}^{4}$ Ω, and the harvester's peak power levels were 34.5 mW at 28.44 Hz, 20 mW at 30 Hz, and 35.1 mW at 32 Hz.

## Future work

When the harvester is tuned as shown in Fig. 21a , it produces low power in between resonance frequencies and peak power at those frequencies. Nonethe–less, by connecting the three floors of beams with an elastic material, this can be improved. That means that the entire array will vibrate, not just certain parts of it when using any of the natural frequencies. A flawless design for a piezoelectric energy harvester will result from this.

## Data Availability

All data generated or analyses during this study are included in this published article.

## List of symbols

${b}_{p}$: Piezoelectric layer thickness
${b}_{s}$: Base material thickness
${c}_{a}$: Damping coefficient for viscous air
${C}_{P}$: The piezoelectric layer's internal capacitance
${c}_{s}$: Composite damping coefficient
$$d$$: Cantilever width
$${e}_{31}$$: Piezoelectric constant
$f$: Frequency of vibration in (Hz)
$I$: Equivalent area moment of inertia
${I}_{t}$: Tip mass's rotational inertia
i: Number of the beam
L: The total length of the cantilever
${L}_{1}$: Length of the first beam
${L}_{2}$: Length of the second beam
${L}_{3}$: Length of the third beam
M(x,t): Moment of internal bending
${m}_{tot.}$: Mass per unit length of the beam
$${M}_{tip.}$$: Tip mass
${N}_{r}^{s}$: Electrical–mechanical coupling function
$$p$$: Power output
${Q}_{rel}(x,t)$: Beam deflection in the transverse direction
${Q}_{b}(x,t)$: Effective transverse base displacement
${Q}_{tot.}\left(x,t\right)$: Total displacement of the harvester
R: Load resistance
t: Thickness of the base material
${t}_{1}$: Height of the first tip mass
${t}_{2}$: Height of the second tip mass
${t}_{3}$: Height of the third tip mass
${v}_{s}$: Series connection coupling term
${w}_{1}$: Width of the first beam
${w}_{2}$: Width of the second beam
${w}_{3}$: Width of the third beam
${w}_{i1}$: The widest width of the beam
${w}_{i2}$: The narrowest width of the beam
${Y}_{s}$: Elastic modulus of the base material
${Y}_{p}$: Elastic modulus of the piezo-material
YI: Equivalent bending stiffness
${z}_{r}$: Term of modal coupling

## Greek symbols

${\rho }_{s}$: Density of the base material
${\rho }_{p}$: Density of the piezoelectric material
${w}_{r}$: The natural frequency of the ${r}{th}$ vibration mode
${\uplambda }_{r}$: System's eigenvalues for mode r
${\alpha }_{r}\left(x\right)$: Mass-normalized eigenfunction
${\upzeta }_{r}$: Modal damping ratio
${\varepsilon }_{33}^{-S}$: Component of permittivity

## References

[1] Yimin, T., Kejian, L. & Zuguang, Z. Comprehensive modelling of a slotless halbach linear generator based wave energy convertor. In *Proceedings of 44th Annual Conference, IEEE Industrial Electronics Society (IECON), Washington DC* (2018).

[2] Lehmann M, Karimpour F, Goudey C, Jacobson P, Alam M (2017). Ocean wave energy in the United States: Current status and future perspectives. Renew. Sustain. Energy Rev..

[3] Erturk A, Hoffmann J, Inman DJ (2009). A piezomagnetoelastic structure for broadband vibration energy harvesting. Appl. Phys. Lett..

[4] Hande A, Polk T, Walker W, Bhatia D (2007). Indoor solar energy harvesting for sensor network router nodes. Microprocess Microsyst..

[5] Anton S, Sodano H (2007). A review of power harvesting using piezoelectric materials (2003–2006). Smart Mater Struct..

[6] Donelan J, Li Q, Naing V, Hoffer J, Weber D, Kuo A (2008). Biomechanical energy harvesting: generating electricity during walking with minimal user effort. Science.

[7] Choi S, Seong M, Kim K (2009). Vibration control of an electrorheological fluid based suspension system with an energy regenerative mechanism. Proc. Inst. Mech. Eng. Part D J. Automob. Eng..

[8] Cassidy, I., Scruggs, J. & Behrens, S. Design of electromagnetic energy harvesters for large-scale structural vibration applications. In *Active and Passive Smart Structures and Integrated Systems 2011*, Vol. 7977 (2011).

[9] Rome L, Flynn L, Goldman E, Yoo T (2005). Generating electricity while walking with loads. Science.

[10] Ali A, Shaukat H, Bibi S, Altabey W, Noori M, Kouritem S (2023). Recent progress in Energy Harvesting Systems for wearable technology. Energy Strategy Rev..

[11] Gammaitoni L, Neri I, Vocca H (2009). Nonlinear oscillators for vibration energy harvesting. Appl. Phys. Lett..

[12] Altabey W, Kouritem S (2022). The new techniques for piezoelectric energy harvesting: Design, optimization, applications, and analysis. Energies.

[13] Liu J, Fang H, Xu Z, Mao X, Shen X, Chen D, Liao H, Cai B (2008). A MEMS-based piezoelectric power generator array for vibration energy harvesting. Microelectron. J..

[14] Moheimani, S. & Fleming, A. Fundamentals of piezoelectricity. In *Piezoelectric Transducers Vibration Control Damping* 9–35 (2006).

[15] Goldschmidtboeing F, Woias P (2008). Characterization of different beam shapes for piezoelectric energy harvesting. J. Micromech. Microengineering.

[16] Hwang S, Jung H, Kim J, Ahn J, Song D, Song Y, Lee H, Moon S, Park H, Sung T (2015). Designing and manufacturing a piezoelectric tile for harvesting energy from footsteps. Curr. Appl. Phys..

[17] Ramalingama U, Gandhi U, Mangalanathanb U, Choi S (2018). A new piezoelectric energy harvester using two beams with tapered cavity for high power and wide broadband. Int. J. Mech. Sci..

[18] Izadgoshasb I, Lim Y, Lake N, Tang L, Padilla R, Kashiwao T (2018). Optimizing orientation of piezoelectric cantilever beam for harvesting energy from human walking. Energy Convers. Manag..

[19] Li M, Jing X (2019). Novel tunable broadband piezoelectric harvesters for ultralow-frequency bridge vibration energy harvesting. Appl. Energy.

[20] Wang L, Ding J, Jiang Z, Luo G, Zhao L, Lu D, Yang X, Ryutaro M (2019). A packaged piezoelectric vibration energy harvester with high power and broadband characteristics. Sens. Actuators A Phys..

[21] Li X, Upadrasht D, Yu K, Yang Y (2019). Analytical modeling and validation of multi-mode piezoelectric energy harvester. Mech. Syst. Signal Process..

[22] Jia Y, Wei X, Xu L, Wang C, Lian P, Xue S, Al-Saadi A, Shi Y (2019). Multiphysics vibration FE model of piezoelectric macro fiber composite on carbon fiber composite structures. Compos. Part B.

[23] Sun S, Tse PW (2019). Modeling of a horizontal asymmetric U-shaped vibration-based piezoelectric energy harvester (U-VPEH). Mech. Syst. Signal Process..

[24] Dhote S, Li H, Yang Z (2019). Multi-frequency responses of compliant orthoplanar spring designs for widening the bandwidth of piezoelectric energy harvesters. Int. J. Mech. Sci..

[25] Asthana P, Khanna G (2019). A broadband piezoelectric energy harvester for IoT based applications. Microelectron. J..

[26] Ma X, Zhao S, Sun X (2019). Design and optimization of a broadband piezoelectric energy harvester. Model. Mech. Mater..

[27] Ghayesha MH, Farokhi H (2020). Nonlinear broadband performance of energy harvesters. Int. J. Eng. Sci..

[28] Lu Z, Chen J, Ding H, Chen L (2020). Two-span piezoelectric beam energy harvesting. Int. J. Mech. Sci..

[29] Li X, Li Z, Huang H, Wu Z, Huang Z, Mao H, Cao Y (2020). Broadband spring-connected bi-stable piezoelectric vibration energy harvester with variable potential barrier. Results Phys..

[30] Qian F, Hajj MR, Zuo L (2020). Bio-inspired bi-stable piezoelectric harvester for broadband vibration energy harvesting. Energy Convers. Manag..

[31] Lia H, Liu D, Wang J, Shang X, Hajj MR (2020). Broadband bimorph piezoelectric energy harvesting by exploiting bending torsion of L-shaped structure. Energy Convers. Manag..

[32] Qian F, Zhou S, Zuo L (2020). Approximate solutions and their stability of a broadband piezoelectric energy harvester with a tunable potential function. Commun. Nonlinear Sci. Numer. Simul..

[33] Kouritem, S. Array of piezoelectric energy harvesters for broadband natural frequency applications. In *Annual Congress of the International Institute of Acoustics and Vibration (IIAV)*, 11–16 (2021).

[34] Cao Y, Cao D, He G, Ge X, Hao Y (2021). Vibration analysis and distributed piezoelectric energy harvester design for the L-shaped beam. Eur. J. Mech./A Solids.

[35] Jiang W, Wang L, Zhao L, Luo G, Yang P, Ning S, Lud D, Lind Q (2021). Modeling and design of V-shaped piezoelectric vibration energy harvester with stopper for low-frequency broadband and shock excitation. Sens. Actuators A Phys..

[36] Fan Y, Ghayesh MH, Lu T (2021). A broadband magnetically coupled bistable energy harvester via parametric excitation. Energy Convers. Manag..

[37] Mohamed K, Elgamal H, Kouritem S (2021). An experimental validation of a new shape optimization technique for piezoelectric harvesting cantilever beams. Alex. Eng. J..

[38] Hani, M., Altabey, W., Abdelnaeem, M. & Kouritem, S. Wideband low-frequency piezoelectric energy harvester with two concentrated proof masses. In *2nd International Conference on Electrical, Computer, Communications and Mechatronics Engineering ICECCME’22* (The Maldives National University, 2022).

[39] Wang J, Li J, Su W, Zhao X, Wang C (2022). A multi-folded-beam piezoelectric energy harvester for wideband energy harvesting under ultra-low harmonic acceleration. Energy Rep..

[40] Kouritem S, Al-Moghazy M, Noori M, Altabey W (2022). Mass tuning technique for a broadband piezoelectric energy harvester array. Mech. Syst. Signal Process..

[41] Hani M, Malkawi D, Hani K, Kouritem S (2023). Genetic algorithm optimization of rainfall impact force piezoelectric sensing device, analytical and finite element investigation. Materials.

[42] Dong L, Zuo J, Wang T, Xue W, Wang P, Li J, Yang F (2022). Enhanced piezoelectric harvester for track vibration based on tunable broadband resonant methodology. Energy.

[43] Hani M, Almomani A, Aljanaideh K, Kouritem S (2022). Mechanical modeling and numerical investigation of earthquake-induced structural vibration self-powered sensing device. IEEE Sens. J..

[44] Shao N, Xu J, Xu X (2022). Experimental study of a two-degree-of-freedom piezoelectric cantilever with a stopper for broadband vibration energy harvesting. Sens. Actuators A Phys..

[45] Zhang B, Li H, Zhou S, Liang J, Gao J, Yurchenko D (2022). Modeling and analysis of a three-degree-of-freedom piezoelectric vibration energy harvester for broadening bandwidth. Mech. Syst. Signal Process..

[46] Fan Y, Ghayesh MH, Lu T, Amabili M (2022). Design, development, and theoretical and experimental tests of a nonlinear energy harvester via piezoelectric arrays and motion limiters. Int. J. Non-Linear Mech..

[47] Kouritem S, El-Gamal H, Mohamed K (2023). Effect of major factors on broadband natural frequency energy harvesting for rectangular and L-shaped cantilever harvesters. Microsyst. Technol..

[48] Wang Z, Wang W, Tang L, Tian R, Wang C, Zhang Q, Liu C, Gu F, Ball AD (2022). A piezoelectric energy harvester for freight train condition monitoring system with the hybrid nonlinear mechanism. Mech. Syst. Signal Process..

[49] Chen J, Bao B, Liu J, Wu Y, Wang Q (2022). Piezoelectric energy harvester featuring a magnetic chaotic pendulum. Energy Convers. Manag..

[50] Li Z, Zhou S, Li X (2022). A piezoelectric–electromagnetic hybrid flutter-based wind energy harvester: Modeling and nonlinear analysis. Int. J. Non-Linear Mech..

[51] Zhao B, Thomsen HR, De Ponti JM, Riva E, Damme B, Bergamini A, Chatzi E, Colombi A (2022). A graded metamaterial for broadband and high-capability piezoelectric energy harvesting. Energy Convers. Manag..

[52] Kouritem S, Altabey W (2022). Ultra-broadband natural frequency using automatic resonance tuning of energy harvester and deep learning algorithms. Energy Convers. Manag..

[53] Kouritem S, Hani M, Beshir M, Elshabasy M, Altabey W (2022). Automatic resonance tuning technique for an ultra-broadband piezoelectric energy harvester. Energies.

[54] Shim H, Sun S, Kim H (2022). On a nonlinear broadband piezoelectric energy harvester with a coupled beam array. Appl. Energy.

[55] Pavlina E, Van Tyne C (2008). Correlation of yield strength and tensile strength with hardness for steels. J. Mater. Eng. Perform..

[56] Anton SR, Erturk A, Inman DJ (2012). Bending strength of piezoelectric ceramics and single crystals for multifunctional load-bearing applications. IEEE Trans. Ultrason. Ferroelectr. Freq. Control.

[57] Erturk A, Inman D (2009). An experimentally validated bimorph cantilever model for piezoelectric energy harvesting from base excitations. Smart Mater. Struct..

[58] Rao SS (1995). Mechanical Vibrations.

[59] Erturk A, Inman DJ (2011). Piezoelectric Energy Harvesting.

[60] Khalili M, Bitenb A, Vishwakarma G, Ahmed S, Papagiannakisc A (2019). Electro-mechanical characterization of a piezoelectric energy harvester. Appl. Energy.

[61] Qi N (2022). Optimization for piezoelectric energy harvesters with self-coupled structure: A double kill in bandwidth and power. Nano Energy.

[62] Lefeuvre DAE, Richard C, Guyomar D (2007). Buck-boost converter for sensorless power optimization of piezoelectric energy harvester. Trans. Power Electron..

[63] Mokhiamar O, Masara D, El-Gamal H (2022). Performance enhancement of multi-modal piezoelectric energy harvesting through parameter optimization. Jordan J. Mech. Ind. Eng..

[64] Galchev T, McCullagh J, Peterson R, Najafi K (2011). Harvesting traffic-induced vibrations for structural health monitoring of bridges. Micromech. Microeng..

[65] Smid P (2009). CNC Programming Handbook.

[66] Overby A (2014). CNC Machining Handbook: Building, Programming, and Implementation.

[67] LaDouceur D (2008). CNC Machining Technology: Volume I: Basics of CNC.

[68] Noori, M. *et al.* Integrating self-powered medical devices with advanced energy harvesting: A review. *Energy Strategy Rev.* **52**, 101328. 10.1016/j.esr.2024.101328 (2024).

[69] Hassan, E., Kouritem, S. A., Amer, F. Z. & Mubarak, R. I. Acoustic energy harvesting using an array of piezoelectric cantilever plates for railways and highways environmental noise. *Ain Shams Eng. J.* **15**(3), 102461 (2024).

[70] Shaukat, H. *et al*. Applications of Sustainable Hybrid Energy Harvesting: A Review *J. of Low Power Electronics and Applications* **13**(4), 62. 10.3390/jlpea13040062 (2023).

[71] Altabey, W. A. *et al.* Advancements in piezoelectric wind energy harvesting *A revi. Results in Eng.* **21**, 101777. 10.1016/j.rineng.2024.101777 (2024).

[72] Ali, A. *et al.* Smart Detecting and Versatile Wearable Electrical Sensing Mediums for Healthcare *Sensors* **23**(14), 6586. 10.3390/s23146586 (2023). 10.3390/s23146586 PMC10384670 37514879

[73] Altabey, W. A. *et al.* Recent progress in energy harvesting systems for wearable technology. *Energy Strategy Rev.* **49**, 101124. 10.1016/j.esr.2023.101124 (2023).

[74] Bani-Hani, M. A. *et al.* Detecting technique of COVID-19 via an optimized piezoelectric sensor. *Jordan J. Mech. Ind. Eng.* **17**(02), 297–307. 10.59038/jjmie/170213 (2023).

[75] Altabey, W. A. & Kouritem, S. A. An overview of the topics of the special issue “The new techniques for piezoelectric energy harvesting: design optimization applications and analysis” *Energies* **16**(8), 3357. 10.3390/en16083357 (2023).

[76] Altabey, W. A. *et al.* Piezoelectric materials: Advanced applications in electro-chemical processes. *Energy Reports* **9**, 4306–4324. 10.1016/j.egyr.2023.03.077 (2023).

[77] Altabey, W. A. *et al.* A Review of the Recent Advances in Piezoelectric Materials Energy Harvester Structures and Their Applications in Analytical Chemistry. *Applied Sciences* **13**(3), 1300. 10.3390/app13031300 (2023).

[78] Kouritem, S. A. *et al.* Energy harvesting from fluid flow using piezoelectric materials: A review. *Energies* **15**(19), 7424. 10.3390/en15197424 (2022).

[79] Kouritem, S. A. *et al.* Structural vibration self-sensing piezoelectric device. https://ieeexplore.ieee.org/abstract/document/10217642 (2023).

